# Transcutaneous Vagus Nerve Stimulation Regulates the Cholinergic Anti-inflammatory Pathway to Counteract 1, 2-Dimethylhydrazine Induced Colon Carcinogenesis in *Albino wistar* Rats

**DOI:** 10.3389/fphar.2019.00353

**Published:** 2019-05-21

**Authors:** Jitendra K. Rawat, Subhadeep Roy, Manjari Singh, Swetlana Guatam, Rajnish K. Yadav, Mohd Nazam Ansari, Sara A. Aldossary, Abdulaziz S. Saeedan, Gaurav Kaithwas

**Affiliations:** ^1^Department of Pharmaceutical Sciences, Baba Saheb Bhimrao Ambedkar Central University Lucknow, Lucknow, India; ^2^Department of Pharmacology, College of Pharmacy, Prince Sattam Bin Abdulaziz University, Al-Kharj, Saudi Arabia; ^3^Department of Pharmaceutical Sciences, King Faisal University, Al-Ahsa, Saudi Arabia

**Keywords:** transcutaneous auricular vagus nerve stimulation, cholinergic anti-inflammatory pathway, colon cancer, acetylcholine, 1, 2-dimethyl hydrazine

## Abstract

The present work was undertaken to study the effects of transcutaneous auricular vagus nerve stimulation (taVNS) on 1, 2-dimethyhydrazine (DMH) induced colon cancer and role of the cholinergic anti-inflammatory pathways (CAP) in the same. Groups of rats were randomly divided into ten groups (*n* = 8). DMH administration was very well apparent for autonomic dysfunction as observed through distorted hemodynamic (electrocardiogram and heart rate variability), increased aberrant crypt foci and flat neoplastic lesions (methylene blue staining, scanning electron microscopy and Hematoxylin and eosin staining). DMH administration was also recorded for per-oxidative damage. taVNS application restored the autonomic function, cellular morphology and curtailed the oxidative damage. DMH application conspicuously inhibited the mitochondrial apoptosis which was restored back after taVNS application, when scrutinized through immunoblotting and quantitative real time polymerase chain reaction studies. taVNS application up-regulated the CAP as perceived through increased expression for α7 nicotinic acetylcholine receptor(α7nAchR) and decreased expression for nuclear factor kappa-ligand-chain-enhancer of activated B cells (NFκBp65), tissue necrosis factor-α and high mobility group box-1 at protein and mRNA levels. All in all, taVNS up-surged the CAP to counteract DMH induced colon carcinogenesis. Among all the stimulation parameters used, taVNS 3 (pulse width-1 ms, frequency-6 Hz, voltage-6 v, duration-240 min) was observed to be the most effective. Since only chemotherapy and surgery are available options for management of CRC, which are troublesome and painful, there is currently no non-invasive method available for management of CRC. Results of the current study affirmed the effectiveness of taVNS against DMH induced colon cancer. The present study established taVNS as a novel and non-invasive approach toward the management of CRC.

## Introduction

Cancer is an abnormal cell division, which begins from mucosal layer of colon and rectum and has the potential to invade adjacent tissues. This is developed due to changeover of normal colonic epithelium to an adenomatous polyp and finally to invasive cancer. Malignant growth of colonic and rectal epithelial cells is a characteristic feature of colorectal cancer (CRC) (An et al., 2016). The risk factors of developing this cancer include inflammatory disease, excessive alcohol consumption, excessive processed food and red meats consumption, physical inactivity, smoking, obesity, diet, polyps, aging, and genetic factors (Haggar and Boushey, 2009; An et al., 2016).

In colon epithelium, the accumulation of genetic and epigenetic changes results in CRC (Lao and Grady, 2011) which is the second most common type of cancer in females (Kuipers et al., 2015). The prolonged exposure to certain risk factors results in imbalanced cellular homeostasis and gene mutation. Tumor suppressor gene and proto-oncogenes play key roles in the development of CRC. Adenomatous polyposis coli gene 14 and RAS gene affect development of CRC during the initial stage. The genotypic expression of some fundamental properties in cell is necessary for carcinogenesis. These properties include: self-sufficiency in proliferation, insensitive nature to antigrowth, elusion to apoptosis, potency of unlimited replication, uninterrupted vascularization, finally invasion and metastasis (Hanahan and Weinberg, 2000). Numerous methods are applied for detection of CRC, including DNA stool test, sigmoidoscopy, colonoscopy, guaiac test, and immunochemical test of stool.

An initial development of adenomatous polyps in the walls of blood vessels and lymph node is a major sign of cancer risk. Surgical removal of affected areas is the first line of treatment of cancer.

After the penetration of cancer into adjacent tissues and the formation of metastasis in blood circulation, chemotherapy is adopted as the second option for the treatment of cancer. Therapy of CRC is difficult when compared to other cancers due to the tremendous rate of metastasis (Granados-Romero et al., 2017). CRC is third most common cancer and second largest cause of cancer related death. CRC is accounting for 700,000 deaths yearly worldwide at this time (Arnold et al., 2017).

Cancer and inflammation are two interconnecting phenomena and studies have established the genetic connection between them (Borrello et al., 2005). Inflammatory responses are essential body defenses and can lead to severe complications (Mantovani and Balkwill, 2001; Tracey, 2002; Mantovani et al., 2008). One of the major regulators of inflammation in colorectal tissues is Ach. The Ach through α7nAchR (ligand gated ion channel) has been demonstrated to have an essential role toward the attenuation of inflammatory responses. The α7nAchR signaling selectively inhibits production of pro-inflammatory cytokines, without having any noticeable effect on anti-inflammatory cytokines (Wang et al., 2003). The production of pro-inflammatory cytokines and other stimuli positively modulates afferent vagus nerve terminals that eventually lead to release of Ach, with consequent activation of α7nAchR. This process inhibits pro-inflammatory cytokine production. This phenomenal arc is known as CAP. It would be appropriate to pen down that α7nAChR has been beforehand reported to be associated with cell death and cancer progression. The role of α7nAChR has been earlier reported in cell invasion and migration in lung cancer cells, pre-cancerous lesions, colon cancer cells and gastric cancer cells, wherein the role of nonspecific agonist of α7nAChR, nicotine was studied on the migration and invasion. Previous studies were particularly based upon the correlation between α7nAChR and nicotine in an isolated system. However, in literature, the composite physiological mechanisms were also taken into consideration to derive a more comprehensive picture (Wei et al., 2011; Yung-Chang et al., 2011; Zhang et al., 2016; Chunxiao et al., 2017). All in all, CAP consists of intricate interactions between the nervous and immune system to regulate inflammatory responses in the colonic mucosa, in which vagus nerve participates as a messenger (Tracey, 2002). In the recent past, studies have reported CAP as a potential chemotherapeutic target in CRC, which can be regulated through the efferent vagus nerve (Pelissier-rota et al., 2013; Sfera et al., 2015). During last decade, taVNS has gained recognition for the treatment of inflammatory disorders like colitis, seizures and depression (Zhao et al., 2012; He et al., 2013; Sun et al., 2013; Kong et al., 2018). In fact, taVNS is approved and nowadays used as well for the clinical management of seizures (Bauer et al., 2016).

Considering the role of taVNS in CAP and clinical success of taVNS, we hypothesize that taVNS could be a potential therapeutic alternative for the clinical management of CRC (Pelissier-rota et al., 2013). Considering that taVNS is a non-invasive clinically validated method, the proposed study holds an upper edge in comparison to the currently available chemotherapeutic options for the management of CRC, which are often troublesome and painful. Henceforth, the present work is an attempt to elaborate the possible mechanisms, implications and possibilities for utilizing taVNS for the management of CRC.

## Materials and Methods

### Drugs and Chemicals

1, 2-dimethylhydrazine (LOT: A0251168) was purchased from Acros organics, NJ, United States. All other chemicals used were of analytical grade and procured from Hi-media Laboratories, Mumbai, India, else otherwise stated in the text.

### Animals

The male *albino wistar* rats (120–140 g) were obtained from the central animal house facility and maintained under standard laboratory conditions, temperature (25 ± 1°C) with 12 h light/dark cycle. Animals were fed with standard animal diet [Chickpeas *(Cicer arietinum)* (30%); DRB (protein and calcium) (10%); Table salt (2%); Husk (20%); (10%); Corn (8%); Refined oil (20%)], and water *ad libitum*.

The study was approved by the institutional animal ethics committee of Sam Higginbottom University of Agriculture, Technology and Science – A Deemed University (approval no. IAEC/SHIATS/PA16III/SJPG17).

### Experimental Protocol

Animals were randomly chosen and grouped in ten groups of eight animals each. Carcinogenicity was induced by weekly injection of DMH (30 mg/kg, s.c) and taVNS was applied at various intensities through the auricular chonchal region.

For taVNS, animals were stimulated with specific stimulating parameters, including pulse width (ms), frequency (Hz), voltage (v) and duration (min). Stimulation started from the 4th day of DMH administration with the exception of taVNS control and dummy VNS groups.

#### Group I (Normal Control)

Animals in this group were administered with 1 mM EDTA–saline (2 ml/kg/day, s.c) for 6 weeks.

#### Group II (taVNS Control)

Animals received taVNS (1.0 ms, 6 Hz, 6 v, and 40 min/week), for 6 weeks without any other treatment.

#### Group III (DMH Control)

1, 2-dimethylhydrazine (30 mg/kg/week, s.c.) was administered for 6 weeks to induce the CRC. DMH was dissolved in 1 mM EDTA-saline and pH 7.0 maintained using NaOH prior to administration (Tanwar et al., 2009; Sanyal et al., 2015).

#### Group IV (DMH + taVNS 1)

Animals received taVNS (1.2 ms, 4 Hz, 6 v, and 33.33 min/week) in conjugation to DMH administration (30 mg/kg/week, s.c.) for 6 weeks.

#### Group V (DMH + taVNS 2)

Animals received taVNS (1.0 ms, 2 Hz, 3 v, and 40 min/week) along with DMH (30 mg/kg/week, s.c.) treatment for 6 weeks.

#### Group VI (DMH + taVNS 3)

Animals received taVNS (1.0 ms, 6 Hz, 6 v, and 40 min/week) in conjugation with DMH (30 mg/kg/week, s.c.) administration for 6 weeks.

#### Group VII (DMH + taVNS 4)

Animals received taVNS (1.4 ms, 6 Hz, 2 v, and 26.66 min/week) along with weekly administration of DMH (30 mg/kg/week, s.c.) for 6 weeks.

#### Group VIII (DMH + taVNS 5)

Animals treated with taVNS (1.6 ms, 1 Hz, 5 v, and 20 min/week) in conjugation with DMH (30 mg/kg/week, s.c.) for 6 weeks.

#### Group IX (Standard Chemotherapy)

Animals treated with Leucovorin followed by 5-Flourouracil (25 mg/kg, i.p. at day 1st, 3rd, 5th, 7th, and 10th after 6-week administration of DMH (30 mg/kg/week, s.c.).

#### Group X (Dummy VNS)

Only stimulating electrodes were placed on the chonchal region of the ear pinna without any other treatment.

**FIGURE 1:**
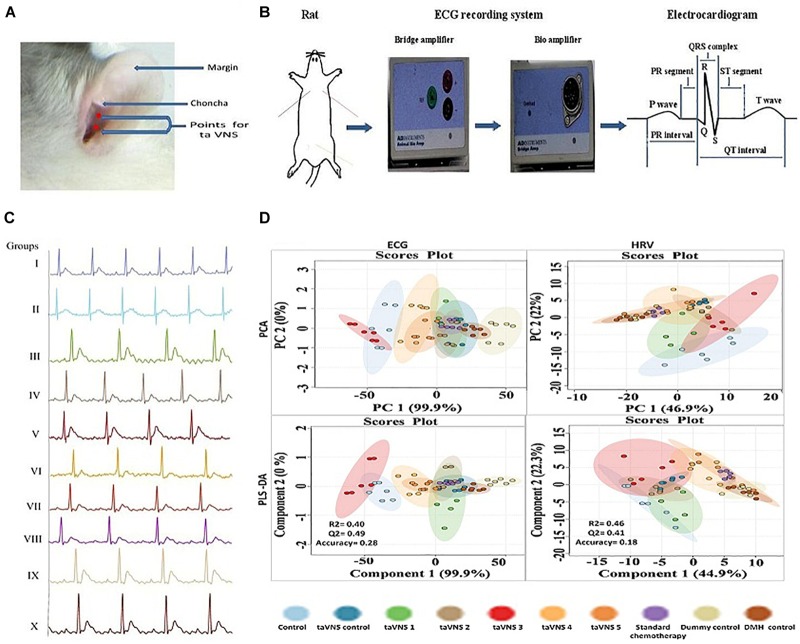
Stimulation, recording, and analysis of the ECG recordings. **(A)** Locations of stimulation points on rat’s auricular surface for taVNS. The auricular choncha is the region of the ear where a major transcutaneous distribution of vagus nerve occurs. The stimulation points for taVNS are located in this region. **(B)** Placement of electrodes and recording of ECG in experimental animals. **(C)** Representative ECG recordings of control, DMH, and taVNS treatments. **(D)** The figure represents for 2D score plots derived from PCA and PLS-DA based analysis for ECG and HRV signals in different groups.

Weight variations between groups were noted throughout experiment for 6 weeks. (Supplementary Table 5 and Figure 1)

All methods were performed as per the guidelines of CPCSEA; Department of Animal Welfare; Government of India.

### taVNS

Animal nerve stimulating electrode (MLA0320, AD Instruments, Australia), consisting of two exposed gold-plated platinum hook electrodes with rounded ends, was used for taVNS. Each electrode had a 2 mm diameter and electrodes were separated from one other by 3 mm.

The animals were anesthetized using a ketamine hydrochloride (100 mg/kg, i.m.) and diazepam (5 mg/kg, i.m.) combination followed by mounting on a wax tray, and electrodes were placed on the auricular chonchal region for stimulation (Rong et al., 2012; Li et al., 2014) (Figure 1A). The electrode leads were connected with power lab system 2/26 (AD Instruments, Australia) for stimulations. ECG and HRV were recorded (Power lab system 2/26, AD Instruments Australia) during stimulation for supervision of hemodynamic variations. To improve electronic conduction, saline was applied on the skin surface. The stimulation started from the 4th day after DMH treatment and continued weekly up to 6 weeks along with DMH. The stimulation parameters are detailed in Supplementary Table 1 (Kong et al., 2012; Li et al., 2014).

On the 42nd day, animals were recorded for ECG and HRV. On the 43rd day blood was collected from retro-orbital plexus and incubated (37°C, 1 h) followed by centrifugation to collect serum. Afterward, the animals were sacrificed under light ether anesthesia. The colon tissue of animals was collected by securing both ends with a surgical suture to prevent drainage of the colonic content (Figure 1B).

### Hemodynamic Changes

The positive and negative gold-plated platinum hook electrodes were placed on the skin on the left and right side of the thorax and the neutral electrode on the skin of the peritoneal region of anesthetized rats. The other ends of the electrodes were connected with power channel (ML-826) and Bio-amplifier (ML-136) for the conversion of analog signals into digital signals. The record ECG signals were analyzed offline, using Lab Chart Pro-8 software. The obtained recording graph was used to calculate ECG and HRV parameters with Lab Chart Pro-8 software. Analysis of hemodynamic raw data started with ensuring the correctness of R wave followed by calculation of heart rate and other ECG parameters. Similarly, the time and frequency-domain parameters for HRV were calculated from the ECG signals using Lab Chart Pro-8 software, following the method elaborated elsewhere (Shintaku et al., 2014; Xiong et al., 2016) (Figure 1B).

### Weight Variation

Weight variation within treatment groups was observed and calculated using the formula I (Mishra et al., 2016).

(1) $$\mathrm{Weight}\;\mathrm{variation}\;(\%)=\frac{\mathrm{Final}\;\mathrm{weight}-\mathrm{Initial}\;\mathrm{weight}}{\mathrm{Final}\;\mathrm{weight}}\times 100(I)$$

### Estimation of pH and Total Acidity

The colonic content was collected and evaluated for pH using pen type pH meter (Hanna Instruments, HI98107). Total acidity was calculated using a previously described procedure (Giri et al., 2015; Kumar et al., 2016).

### ACF

Longitudinally opened colonic tissue was washed with normal saline and fixed by placing it between two layers of whatman paper for 24 h in 10% formalin. Subsequently, tissue was stained with 2% methylene blue and visualized through the light microscope. The ACF was visualized and counted in five independent frames using the method elaborated previously by our laboratory (Bird, 1987; Mishra et al., 2016).

### Morphological Evaluation

Scanning electron microscope (JEOL-JSM-6490LV) was used to study the morphological changes on the colonic mucosa. Samples were prepared by fixation of tissues in 2.5% glutaraldehyde for 6 h at 4°C. Tissues were washed with 0.1 M phosphate buffer; post fixed and dehydrated using the method described elsewhere. The tissues were mounted on the aluminum stub with adhesive tape and visualized at magnification of 500X and 2000X (Cooke et al., 1984; Sammi et al., 2018).

### Histopathology

Hematoxylin and eosin staining was used to study the histopathology of colonic tissues. The tissues were fixed overnight in paraformaldehyde with subsequent dehydration using isopropanol and xylene. Dehydrated tissues were fixed in paraffin wax and sectioned (0.5 mm) using a microtome. Sections were stained with H&E, and visualized under the digital biological microscope at a magnification of 40X (N120, BR-Biochem Life Sciences, New Delhi, India) (Serafino et al., 2014). Quantification of H&E staining and SEM was performed by using Image J software (Jensen, 2013).

### Antioxidant Markers

The colon tissues (10% w/v) were homogenized in 0.15% KCl and centrifuged at 10,000 rpm. The supernatant was used for biochemical estimations for TBAR’s, SOD, catalase, GSH, and PC using previously established methods at our laboratory (Gupta et al., 2014; Kumar et al., 2014; Raj et al., 2014; Gautam et al., 2016).

### Western Blotting

The colon tissue was lysed in the RIPA lysis buffer to obtain total protein lysate and quantified using Bradford’s reagent. The proteins were resolved through 12.5% sodium dodecyl sulfate-polyacrylamide gel electrophoresis and transferred to polyvinylidene difluoride membrane (IPVH 00010 Millipore, Bedford, MA United States) using a method established in our laboratory (Roy et al., 2017).

Following the transfer, the membrane was blocked with solution of bovine serum albumin and skimmed milk in TBST (3%) for 3 h. The blocked membrane was incubated overnight with primary antibodies for Bcl-xl (MA-5-15142), B cell lymphoma- 2 (Bcl-2) (SC-7382), BAX (SC-23959), BAD (SC-8044), VDAC (SC-390996), cytochrome-c (SC-13561), Apaf-1 (SC-65891), procaspase-9 (SC 73548), HMGB-1 (SC-56698), α7nAchR (SC-5544), NFκBp65 (MA5-1616), and TNF-α (SC-1350) at 4°C. After incubation with primary antibody, the membrane was washed thrice with TBST and once with TBS. Subsequently, the membrane was incubated with the HRP conjugated secondary antibody, including anti-rabbit (SC-2030), anti-goat (SC-2020), anti-mouse (31430, Pierce Thermo Scientific, United States) (1:5000 dilutions) for 3 h.

The membrane was developed by using enhanced chemiluminescence substrate (Western Bright ECL HRP substrate, Advansta, Melanopark, CA, United States) in gel dock system after three and single washings with TBST and TBS, respectively. The protein bands were quantified by densiometric digital analysis using Image J software. β-actin (MA5-15739-HRP) was used as a standard reference (Roy et al., 2017).

### qRT-PCR

Primer quest, a tool from the Integrated DNA Technologies website^1^, was used for online primer designing in real time. Size of the amplicon was kept between 100 and 200 base pairs. Guanine-cytosine ratio was kept above 50% and melting temperature was kept between 58 and 62°C. The sequences of forward and reverse primers used for qRT-PCR are detailed in Supplementary Table 2 (Liu et al., 2004).

Total RNA was extracted from colon tissues using trizol reagent according to manufacturer instructions. Tissues were meshed in 250 μl trizol reagent by using micro pestle and volume was made up to 1 ml followed by addition of 200 μl chloroform and vortexing for 5 min.

Tissue suspension was centrifuged at 14,000 rpm, 4°C for 15 min. The aqueous phase was separated and transferred to a fresh vial, with subsequent centrifugation at 14000 rpm for 10 min. The pellets were washed twice with chilled ethanol and dissolved in 15 μl of 1% diethyl polycarbonate water. The samples were quantified through nanodrop (Qua Well Q5000).

1 μg of tissue RNA was used for cDNA synthesis, which was used as a template for qRT-PCR reaction using the method described previously by us. β actin was used as a house keeping control (Roy et al., 2017).

### Assay for Caspase 3 and Caspase 8

Fluorometric assays for caspase 3 and 8 were performed in amber colored 96 well plate using the protocol provided by the manufacturer. The equal volume of serum taken from all treatment groups was diluted with reaction buffer followed by addition of dithiothriol to a final concentration of 10 mM. 5 μl of IETD-AFC/DEVD-AFC substrate was added to the reaction mixture and incubated for 1 h at 37°C. The formed free AFC levels were measured in a plate reader at 400 nm excitation and 505 emissions. The results were expressed as fluorescence units/mg of protein (Kim et al., 2003; Brown et al., 2015).

### Statistical Analysis

Results are presented as mean ± SD and analyzed by one-way ANOVA followed by Bonferroni test for the possible significance identification between the various groups. ^a^*p* < 0.05, ^b^*p* < 0.01, ^c^*p* < 0.001 were considered statistically significant. Statistical analysis was performed using Graph Pad Prism software (5.02).

Electrocardiogram and HRV were analyzed using Metabo Analyst. Metabo Analyst is a web-based data analysis tool. In this, obtained results are in tabular as well as in the graphical format, easy to understand. Metabo Analyst can be used for various analyses including biomarker analysis and pathway analysis but is here used for statistical analysis. Raw data was saved in the comma separated values and uploaded as spectral bins followed by data integrity check, data filtering, and normalization of data (pareto scaling). Finally, multivariate analysis of data was performed using principal component analysis and partial least squares – discriminant analysis method (Xia et al., 2009).

## Results

### Hemodynamic Studies

The DMH treatment was very well manifested for increase in RR interval (0.20 ± 0.01s), heart rate (380.1 ± 29.96 bpm), QRS interval (0.02 ± 0.001 s), P(0.04 ± 0.003 mv), and Q (0.20 ± 0.01 mv) wave amplitude along with decrease in QT interval (0.07 ± 0.007 s), JT interval (0.05 ± 0.003 s), QTc complex (0.15 ± 0.01 s) and R wave amplitude (1.35 ± 0.12 mv) (Figure 1C, Supplementary Table 3, and Supplementary Figure 2). The principal component analysis and partial least squares – discriminant analysis of the ECG signals revealed that taVNS 3, 4, and 5 could favorably regulate the ECG signals toward control. The HRV analysis revealed significant curtailment of average RR (157.80 ± 9.15 ms), median RR (158.8 ± 10.26 ms), low frequency (1.2 ± 0.10 μs^2^), high frequency (9.10 ± 0.81 μs^2^), and low frequency/high frequency (0.13 ± 0.01) in DMH treated animals (Supplementary Table 4 and Supplementary Figure 3). taVNS 2 and 3 could favorably curtail the deleterious effects of DMH (Figure 1D). When compared with standard chemotherapy, treatment with taVNS more favorably restored hemodynamic changes toward normal control (Supplementary Figures 2, 3).

### pH, Total Acidity, Percentage Weight Variation, and ACF Count

When scrutinized on the account of physiological parameters, DMH treatment showed significant decrease in weight (-14.17 ± 1.32%) (Supplementary Figure 1), non-consequential decrease in pH (6.31 ± 0.56), with increase in total acidity (127.96 ± 8.6 mEql^-1^) and ACF (56 ± 2.29NoS). taVNS regulated the weight variation and pH significantly. The total acidity and ACF count were favorably regulated more profoundly by taVNS3. When compared with standard chemotherapy, taVNS resulted in better regulation of weight variation (all taVNS groups), pH (taVNS 1 and 2) and total acidity (taVNS 1) toward control (Table 1, Supplementary Table 5, and Supplementary Figure 1). The taVNS treatment could not regulate the ACF when compared with standard chemotherapy against DMH induced colon carcinogenesis (Table 1).

**Table 1:** Effects of taVNS on weight variation, pH, total acidity, and ACF count.

	Weight variation (%)	pH	Total acidity (mEql^-1^)	Aberrant crypts(NoS)
Control	21.22 ± 1.75	6.97 ± 0.62	95.97 ± 4.5^c^	11.6 ± 0.87^c^
taVNS control	22.4 ± 1.43^c^	6.98 ± 0.59	95.06 ± 5.6^c^	16.4 ± 1.13^c^
DMH control	-14.17 ± 1.32	6.31 ± 0.56	127.96 ± 8.6	56 ± 2.29
taVNS 1	18.65 ± 1.56^c^	7.01 ± 0.61	93.97 ± 7.56^c^	21.4 ± 1.72^c^
taVNS 2	20.95 ± 1.62^c^	7.00 ± 0.63	94.74 ± 6.7^c^	22 ± 0.96^c^
taVNS 3	24.42 ± 1.95^c^	6.98 ± 0.56	95.45 ± 8.12^c^	28 ± 1.64^c^
taVNS 4	26.8 ± 2.18^c^	6.91 ± 0.61	100.97 ± 7.3^c^	30.2 ± 1.12^c^
taVNS 5	30 ± 2.31^c^	6.85 ± 0.62	103.36 ± 6.95^c^	38 ± 2.70^c^
Standard chemotherapy	12.4 ± 0.98^c^	6.99 ± 0.57	94.37 ± 5.72^c^	13.8 ± 1.12^c^
Dummy control	24.84 ± 2.12^c^	6.92 ± 0.54	100.73 ± 7.23^c^	17.4 ± 0.79^c^


*(Values are presented as Mean ± SD), each group contains eight animals. Comparisons were made on the basis of the one-way ANOVA followed by Bonferroni multiple tests. All groups were compared to the DMH control group (^*a*^*p* < 0.05, ^*b*^*p* < 0.01, ^*c*^*p* < 0.001). I-Control, II-taVNS control, III-DMH control, IV-taVNS1, V-taVNS 2, VI-taVNS 3, VII-taVNS 4, VIII-taVNS 5, IX-Standard chemotherapy, X-Dummy control.*

### Anticancer Assay

When accounted for the oxidative stress markers, the DMH treatment upsurged TBARs (187.82 ± 6.78 nM of MDA/μg of protein) and PC (106.68 ± 10.82 nM/ml unit) in comparison to control. The SOD (0.004 ± 0.001 unit of SOD/mg of protein) and catalase (1.67 ± 0.086 nM of H_2_O_2_/min/mg of protein) levels were down-regulated with a significant increase in GSH (0.68 ± 0.07 mg %) in DMH treated animals. taVNS 2 and 3 embarked upon a more favorable regulation for TBARs and PC, respectively. taVNS 2 and 3 regulated the GSH, SOD and catalase as well (Table 2). When compared to the effects of taVNS and standard chemotherapy against DMH induced colon carcinogenesis, taVNS was more effective in regulating the TBARs (all taVNS treatments), catalase (all taVNS treatments), and GSH (taVNS 4).

**Table 2:** Effects of taVNS on biological markers of oxidative stress against DMH induced colon carcinogenesis.

	Control	taVNS control	DMH control	taVNS 1	taVNS 2	taVNS 3	taVNS 4	taVNS 5	Standard chemotherapy	Dummy control
TBARs (nM of MDA/μg of protein)	101.06 ± 4.81	140 ± 14.90^c^	187.82 ± 6.78	124.35 ± 6.11^c^	104.70 ± 1.95^c^	126.49 ± 17.39^c^	131.75 ± 3.24^c^	124.35 ± 4.50^c^	136.36 ± 22.86^c^	146.98 ± 9.81^b^
Glutathione (mg %)	0.36 ± 0.01^c^	0.47 ± 0.02^c^	0.68 ± 0.01	0.61 ± 0.12^c^	0.38 ± 0.01^c^	0.45 ± 0.06^c^	0.32 ± 0.06^c^	0.36 ± 0.01^c^	0.35 ± 0.03^c^	0.38 ± 0.01^c^
Catalase (nM of H_2_O_2_/min/mg of protein)	1.75 ± 0.1	2.18 ± 0.09	1.67 ± 0.09	2.13 ± 0.15^c^	1.81 ± 0.03	2.09 ± 0.03^c^	1.76 ± 0.02	1.83 ± 0.02^b^	2.15 ± 0.08^c^	1.69 ± 0.08
SOD (unit of SOD/mg of protein)	0.03 ± 0.002^c^	0.04 ± 0.002^c^	0.004 ± 0.001	0.02 ± 0.003^c^	0.03 ± 0.001^c^	0.03 ± 0.002^c^	0.02 ± 0.001^c^	0.04 ± 0.002^c^	0.04 ± 0.004^c^	0.02 ± 0.01^c^
Protein carbonyl (nM/ml unit)	34.31 ± 3.74	50.89 ± 8.65^c^	106.68 ± 10.8	53.90 ± 6.02^c^	74.99 ± 3.69^c^	58.78 ± 7.63^c^	102.21 ± 2.59	62.63 ± 3.50^c^	40.30 ± 1.95^c^	44.14 ± 5.45^c^


*(Values are presented as Mean ± SD), each group contains eight animals. Comparisons were made on the basis of the one-way ANOVA followed by Bonferroni multiple tests. All groups were compared to the DMH control group (^*a*^*p* < 0.05, ^*b*^*p* < 0.01, ^*c*^*p* < 0.001). I-Control, II-taVNS control, III-DMH control, IV-taVNS1, V-taVNS 2, VI-taVNS 3, VII-taVNS 4, VIII-taVNS 5, IX-Standard chemotherapy, X-Dummy control.*

Treatment with taVNS resulted in positive regulation of PC and GSH against DMH control but was comparatively less effective as standard chemotherapy.

### Morphological and Histopathological Evaluation

**FIGURE 2:**
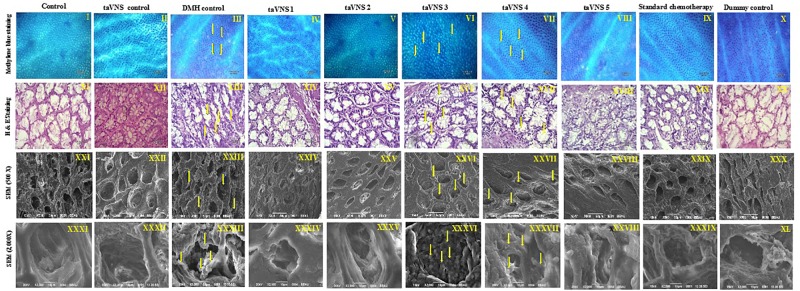
Methylene blue staining, H&E staining, and scanning electron microscopy of colon tissue. I–X – Methylene blue staining of colon tissue; XI–XX – represents H&E staining of colon tissue; XXI–XXX – represents scanning electron microscopy of colon tissue (500X) and XXXI–XL – represents scanning electron microscopy of colon tissue (2000X) depicts presence of small neoplastic lesions on mucosal tissue of DMH control. The arrows represent ACF formation and neoplastic lesions.

The colonic tissue was evaluated morphologically through methylene blue staining, H&E staining, and SEM. After methylene blue staining of the colonic mucosa of DMH treated animals, dark-blue stained cells were visualized representing ACF. The ACF in the colonic mucosa of the DMH treated animals was also evident through H&E staining and SEM. The SEM images of DMH treated animals were besides apparent for small neoplastic lesions (Figure 2 – XXXIII). taVNS 3 and 4 curtailed the ACF formation and normalized the histopathological architecture more favorably. The formations of neoplastic lesions were also subsided after the taVNS application in general and profoundly by taVNS 3 and 4 (Figure 2, Supplementary Figure 4, and Supplementary Table 6).

### Western Blotting

Proteins extracted from individual groups were subjected to immunoblotting assay for anti-apoptotic (Bcl-2 and Bcl-xl), pro-apoptotic (BAX and BAD) proteins along with downstream markers of the apoptotic pathway (VDAC, cytochrome-c, Apaf-1 and procaspase-9).

After treatment with DMH, the expression of anti-apoptotic proteins (Bcl-2 and Bcl-xl) was increased. The expression of pro-apoptotic protein BAX was decreased while the BAD was increased. The taVNS application resulted in down-regulation of Bcl-2, with mixed effects on expression of Bcl-xl was noted. taVNS 1, 2 and 3 resulted in Bcl-xl up-regulation while taVNS 4 and 5 resulted in down-regulation. In response to DMH treatment, expression of BAX decreased while BAD increased. taVNS resulted in down-regulation of BAX and up-regulation of BAD. All in all, taVNS was recorded to have more favorable regulation of pro-and anti-apoptotic proteins, when compared to DMH treated animals.

1, 2-dimethylhydrazine administration was also evident for increased expression for VDAC, Apaf-1 and procaspase-9 with the concomitant decrease in cytochrome-c expression.

**FIGURE 3:**
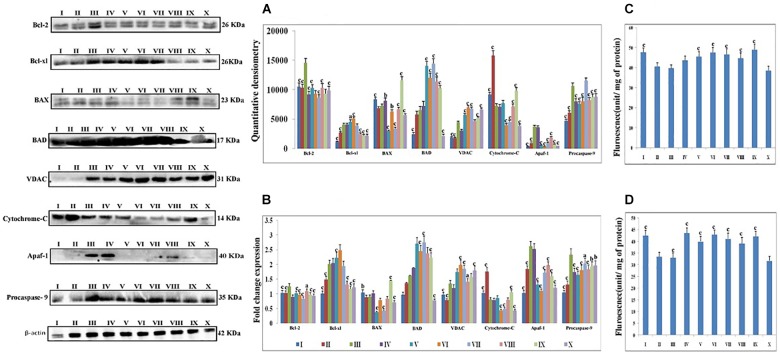
Effect of taVNS on mitochondrial apoptosis. The details of the groups for western blot analysis and caspase assay are as follows: I-Control, II-taVNS control, III-DMH control, IV-taVNS1, V-taVNS 2, VI-taVNS 3, VII-taVNS 4, VIII-taVNS 5, IX-Standard chemotherapy, X-Dummy control. Specific treatments for particular groups mentioned in Supplementary Table 1. **(A)** Proteins were extracted from individual groups and subjected to immunoblotting of pro-apoptotic (BAX and BAD) and anti-apoptotic (Bcl-2 and Bcl-xl) markers along with downstream markers of apoptotic pathway (VDAC, cytochrome-c, Apaf-1, and procaspase 9). After treatment with DMH the anti-apoptotic proteins (Bcl-2 and Bcl-xl) expression was increased. The expression of pro-apoptotic protein BAX decreased while BAD increased. The taVNS treatment results in restoration of anti-apoptotic and pro-apoptotic proteins with exception of BAD. When observed, the expression of markers of mitochondrial apoptosis (VDAC, cytochrome c, Apaf-1, and procaspase 9) treatment with DMH results in increased expression of VDAC, Apaf-1, and procaspase 9 along with decreased expression of cytochrome c. Treatment with taVNS resulted in increased expression of cytochrome c and decreased expression of procaspase 9. **(B)** The outcomes from the immunoblotting assay were confirmed through qRT-PCR studies by scrutinizing the respective phenotypes of pathway associated proteins. The mRNA expressions of the above mentioned protein were also in line with the findings of immunoblotting assay. β-actin was used as loading control in immunoblotting and qRT-PCR assay. **(C,D)** Represent the levels of caspase 3 and 8, respectively. The activity of caspase was detected by commercial fluorescence based assay. The caspase 3 and caspase 8 levels were decreased after DMH treatment and taVNS (particular taVNS 3) up-regulated the same. Each experiment was performed in triplicate. Values are presented as mean ± SD. Comparisons were made by the one-way ANOVA followed by Bonferroni multiple test. All groups were compared to the DMH treated group (^a^*p* < 0.05, ^b^*p* < 0.01, ^c^*p* < 0.001).

Transcutaneous vagus nerve stimulation down-regulated the VDAC (taVNS 1), Apaf-1 and procaspase-9 (except taVNS 4) expression (Figure 3A). taVNS favorably regulated the expression of VDAC (taVNS 1), Apaf-1 (taVNS 1 and 2), and procaspase-9 (except taVNS 4) (Figure 3A). DMH treatment was also evident for decreased levels of caspase 3 and 8, which were up-regulated after taVNS application (Figure 3C,D).

**FIGURE 4:**
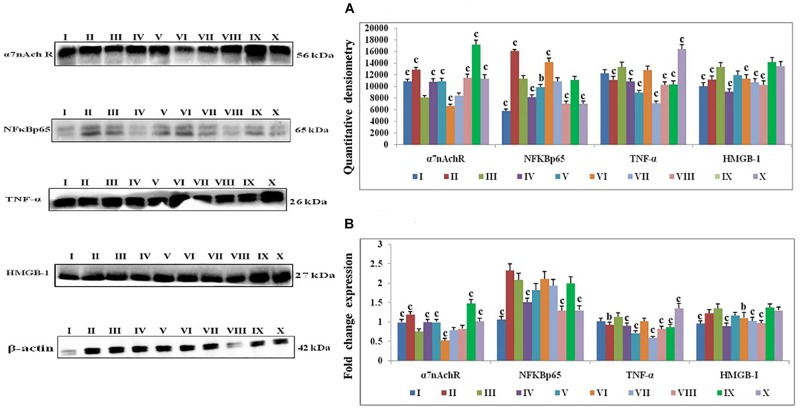
Effect of taVNS on cholinergic anti-inflammatory pathway in DMH induced colon carcinogenesis. The details of groups as follows for western blot analysis – I-Control, II-taVNS control, III-DMH control, IV-taVNS 1, V-taVNS 2, VI-taVNS 3, VII-taVNS 4, VIII-taVNS 5, IX-Standard chemotherapy, X-Dummy control. Specific treatments for individual groups mentioned in Supplementary Table 1. **(A)** Immunoblotts for α7nAchR, NFκB, TNF-α, and HMGB-1 for various groups. The expression of α7nAchR was decreased while NFκB, TNF-α, and HMGB-1 increased after DMH treatment. The taVNS treatment resulted in increased expression of α7nAchR and curtailment of inflammatory markers (NFκB, TNF-α, and HMGB-1). **(B)** The mRNA expressions of the above mentioned protein were also in line with the findings of immunoblotting assay. β-actin was used as loading control in immunoblottingand qRT-PCR assay. Each experiment was performed in triplicate. Values are presented as mean ± SD. Comparisons were made by the one-way ANOVA followed by Bonferroni multiple test. All groups were compared to the DMH treated group (^a^*p* < 0.05, ^b^*p* < 0.01, ^c^*p* < 0.001).

1, 2-dimethylhydrazine treatment decreased the α7nAchR expression with up-surged expression of NFκBp65, TNF-α, and HMGB1. Majority of taVNS treatments down-regulated the expression for NFκBp65 (except taVNS3), TNF-α, HMGB-1and up-regulated the α7nAchR expression (except taVNS 3) (Figure 4A). On considering standard chemotherapy, the majority of taVNS treatments resulted in better responses in regulation of CAP associated proteins with the exception of α7nAchR.

### qRT-PCR

The fold change of gene expressions for a particular protein was validated through qRT-PCR. The qRT-PCR assay was used for the affirmation of genetic phenotypes for the protein markers associated with mitochondrial apoptotic pathway and CAP. Results of qRT-PCR were found in line with immunoblotting assay. Significant fold change of gene expression was noted in response to taVNS application. When considering mitochondrial apoptotic pathway, gene expression and protein expression of Bcl-2 and BAX were decreased and BAD expression was increased after taVNS application. taVNS exerted mixed effects for gene expression of Bcl-xl, taVNS 1,2 and 3 up-regulated while taVNS 4 and 5 down-regulated the expression of Bcl-xl.

All in all, taVNS positively modulated the gene expression for pro-and anti-apoptotic genes as compared to DMH application. DMH treatment was evident for increased expression for VDAC, Apaf-1, and procaspase-9 with collateral decreased cytochrome-c gene expression. qRT-PCR affirmed down-regulation of VDAC (taVNS 1), Apaf-1 and procaspase-9 with concomitant up-regulation of cytochrome-c gene expression in response to taVNS treatment (Figure 3B). qRT-PCR studies validated the effects of taVNS on CAP associated proteins. Toxicant treatment decreased the α7nAchR gene expression along with increased gene expression of NFκBp65, TNF-α, and HMGB-1. All taVNS treatments decreased the expression for NFκBp65, TNF-α, HMGB1 and increased the α7nAchR expression (except taVNS 3) (Figure 4B).

## Discussion

Autonomic dysfunction is reported in most advanced cancers (Walsh and Nelson, 2002). HRV is a functional biomarker of autonomic nervous system. HRV is a measurement of autonomic nervous system through sympathetic and parasympathetic modulations of cardiac functions. Vagus nerve is considered as a main component of parasympathetic nervous system and previous literature established the role of vagus nerve in cancer prognosis (De Couck et al., 2018).

Antecedent studies reported significant lowering of HRV in cancer patients as compared to healthy individuals (Palma et al., 2016; Kloter et al., 2018). Previous studies have also endorsed distorted ECG and lower HRV in clinical cases and preclinical cancer models, which was very well apparent in DMH treated animals (Guo et al., 2013; Sammi et al., 2018). Recent studies reported modulatory action of taVNS on HRV and thus parasympathetic nervous system (De Couck et al., 2016; Murray et al., 2016; Ylikoski et al., 2017). Further studies are needed to determine the effect of taVNS on HRV of clinical patients of cancer.

Transcutaneous vagus nerve stimulation 3 was recorded to have the most significant effect upon the ECG among all the stimulation methods used. taVNS 3 also up-regulated the time and frequency-domain parameters for HRV. It would be appropriate to remark that a decrease in HRV has emerged as a non-invasive marker for autonomic dysfunction in cancer sufferers (De Couck and Gidron, 2013; Palma et al., 2016; Kloter et al., 2018). In the instant study, taVNS 3 was recorded to regulate autonomic dysfunction more favorably in comparison to other stimulation methods used in the study. The reduction in weight in DMH treated animals could be attributed to the increased biological demands of the body due to fast growing tumor cells. taVNS 3 was also recorded to favorably regulate pH, total acidity and weight variation in the DMH treated animals.

Cancer development is a multistep process. Excessive oxidative stress is involved in all stages of cancer development (Skrzydlewska et al., 2001). Increased levels of TBAR’s, PC, and GSH along with reduced levels of catalase and SOD were noted in response to DMH treatment.

Thiobarbituric acid reactive substances and PC are the sensitive and reactive markers of membrane damage and are stable products of lipid and protein peroxidation (Shukla et al., 2014). In the present study, consequential increases in TBAR’s and PC levels of DMH treated animals were recorded, supporting the involvement of reactive oxygen species in the progression of colon cancer. taVNS 2 and 3 resulted in momentous reductions in lipid and protein peroxidation as confirmed by the decrease in TBAR’s and PC. SOD and catalase are the endogenous free radical scavengers. It would be appropriate to mention that both catalase and SOD work in tandem and form a defensive team for protection against injurious free radicals. SOD scavenges superoxide radical to yield H_2_O_2_ and molecular oxygen with subsequent catabolism of H_2_O_2_ by catalase to water and molecular oxygen (Singh et al., 2008; Kaithwas et al., 2011). The decreased levels of SOD and catalase are reported and were recorded after DMH treatment in the present study. This decrease in of SOD and catalase could be attributed to increased utilization in response to oxidative stress. taVNS 2 and 3 resulted in restoration of the SOD and catalase, confirming the say of taVNS in colon carcinogenesis.

Morphological characterization is a well-established and affirmative method for cancer diagnosis (Shia et al., 2017). When studied morphologically, the colonic mucosa of the DMH treated animals was conspicuous with the presence of ACF. The presence of ACF was very well validated using methylene blue staining, SEM, and H&E staining and is in line with previous literature (Mishra et al., 2016).When observed more closely, small neoplastic lesions were also perceived in the colonic tissue. However, taVNS markedly reduced the ACF count, with more profound effect by taVNS 3 and 4. All in all, the proposed taVNS therapy restored the colonic architecture close to normal. It would not be out of place to mention that the neoplastic lesions as observed after DMH administration was also subsided after taVNS 3 and 4.

Carcinogenesis is a complex phenomenon, and it is settled scientific theory that loss of apoptosis is the mediator for carcinogenesis (Lowe and Lin, 2000). Apoptotic pathway is majorly governed by mitochondria under the direct control of several proteins. Categorically mitochondrial apoptotic proteins are either pro-apoptotic (BAX and BAD) or anti-apoptotic (Bcl-2 and Bcl-xl) in nature (Gross et al., 1999). Treatment with DMH was very well apparent for progression of colon carcinogenesis, through up-regulated expression for anti-apoptotic (Bcl-2 and Bcl-xl) signals and down-regulated expression for pro-apoptotic (BAX) signals, with more profound effect by taVNS 5.

Progression of apoptosis is further mediated through the release of cytochrome-c from mitochondria and formation of apoptosomes, causing decreased expression for cytochrome-c and Apaf-1 along with cleavage of procaspase-9 (Estaquier et al., 2012). Treatment with taVNS down-regulated cytochrome-c, Apaf-1, and procaspase-9 expressions in comparison to DMH. The findings from the immunoblotting assay were further affirmed through qRT-PCR studies through scrutinizing the respective phenotypes. It would not be out of place to mention that the above findings are in line with previous reports (Youle and Strasser, 2008; Estaquier et al., 2012). taVNS was reported to counteract the deleterious effects of DMH by inducing apoptosis, with more pronounced effect by taVNS 5.

The study was further extended to scrutinize the serum level of caspase 3 and 8 (effector caspases for execution of apoptosis). DMH treatment decreased the levels of caspase 3 and 8, which is in line with previous reports (Brown et al., 2015; McIlwain et al., 2015). Concomitant taVNS application upsurged the levels of caspase 3 and 8. The above findings clearly confirm the induction of mitochondrial apoptosis with taVNS application.

All in all, taVNS application counteracted the carcinogenic effects of DMH, by up- regulating mitochondrial apoptosis. Henceforth, it becomes imperative for us to study the effect of taVNS on CAP as per our original hypothesis.

**FIGURE 5:**
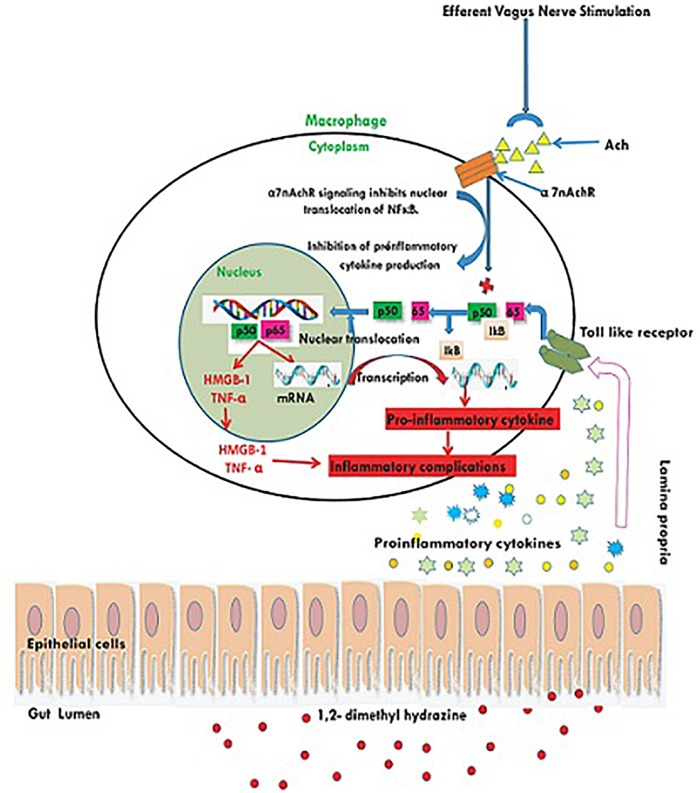
Schematic representation for the effect of taVNS on CAP. Inflammatory/toxic substances invoke series of pro-inflammatory signals to bind with toll like receptors on the cellular membranes. Binding to the toll like receptors mediates the translocation of NFκB to nucleus and release of pro-inflammatory mediators. taVNS can facilitate the Ach release to act upon the α7nAchR receptor, which in turn inhibits the translocation of NFκB to nucleus and therefore curtails the inflammatory signaling.

1, 2-dimethylhydrazine administration curtailed the α7nAchR expression at protein and mRNA level, which was up-regulated after taVNS application. The up-regulation of α7nAchR expression after taVNS application is a clear indication of induction of CAP. The up-regulation of CAP was further substantiated by the observation that taVNS application curtailed the expression of inflammatory markers (NFκBp65 and TNF-α). It would be appropriate to mention that taVNS 3, 4 and 5 imparted more favorable effect upon α7nAchR, NFκBp65, and TNF-α respectively. One of the major executors for inflammatory cascade regulated by NFκBp65 and TNF-α is HMGB-1, and the same was up-regulated after the DMH treatment. It is widely reported in literature that CAP curtails NFκBp65 and subsequently downstream mediator HMGB-1, which was very well evident after taVNS (Brem et al., 2007; Garzoni et al., 2013). It would not be out of place to mention that taVNS 4 and 5 were equipotent to curtail the expression for HMGB-1. The above findings are clear endorsement for up-regulation of CAP mediated through α7nAchR signaling as a consequence of taVNS (Figure 5).

The authors would like to conclude that taVNS imparted a significant effect upon DMH induced colon carcinogenesis mediated through CAP. It is furthermore submitted that taVNS could in addition be explored as a strategy in the management of colon carcinogenesis, however, the same needs to be validated through clinical studies. Moreover, the use of taVNS as a complimentary therapy for colon carcinogenesis is under investigation in our laboratory.

## Ethics Statement

The study was approved by the institutional animal ethics committee of Sam Higginbottom University of Agriculture, Technology and Science – A Deemed University (approval no. IAEC/SHIATS/PA16III/SJPG17). All methods were performed as per the guidelines of CPCSEA; Department of animal welfare; Government of India.

## Author Contributions

JR carried out the bench work. SR performed western blot studies. RY performed the methylene blue staining. MS performed the SEM. SG performed histopathological studies. MA and SA performed the statistical analysis. AS compiled the data. GK perceived the idea, designed and supervised the overall study, prepared and proofread the final manuscript.

## Conflict of Interest Statement

The authors declare that the research was conducted in the absence of any commercial or financial relationships that could be construed as a potential conflict of interest.

## Footnotes

1 http://www.idtdna.com

## Abbreviations

ACF: aberrant crypt foci
Ach: acetylcholine
Apaf-1: apoptotic protease activating factor-1
Bcl-2: B cell lymphoma-2
CAP: cholinergic anti-inflammatory pathway
CRC: colorectal cancer
DMH: 1, 2-dimethylhydrazine
ECG: electrocardiogram
GSH: glutathione
H_2_O_2_: hydrogen peroxide
HMGB-1: high mobility group box-1
HRV: heart rate variability
H&E staining: hematoxylin and eosin staining
NFκB: Nuclear factor kappa-ligand-chain-enhancer of activated B cells
PC: protein carbonyl
qRT-PCR: quantitative real-time polymerase chain reaction
SEM: scanning electron microscope
SOD: superoxide dismutase
TBAR’s: thiobarbituric acid reactive substances
TNF-α: tissue necrosis factor-α
taVNS: transcutaneous vagus nerve stimulation
VDAC: voltage-dependent anion channel
α7nAchR: α7 nicotinic acetylcholine receptor
