# Supplementary material for: Transcutaneous Vagus Nerve Stimulation Regulates the Cholinergic Anti-inflammatory Pathway to Counteract 1, 2-Dimethylhydrazine Induced Colon Carcinogenesis in Albino wistar Rats
Source: Front Pharmacol. 2019 May 21;10:353. doi: 10.3389/fphar.2019.00353 (PMC6536668; doi:10.3389/fphar.2019.00353)
Supplement: Supplementary file 1 [file Data_Sheet_1.PDF]

Transcutaneous vagus nerve stimulation regulates cholinergic anti-inflammatory pathway to counteract 1, 2-dimethyl hydrazine induced colon carcinogenesis in *albino wistar* rats

Jitendra K Rawat[1], Subhadeep Roy[1], Manjari Singh[1], Swetlana Guatam[1], Rajnish K Yadav[1], Mohd Nazam Ansari[2], S. A. Aldossary[3], Abdulaziz S Saeedan[2], Gaurav Kaithwas[1,*]

1. Department of Pharmaceutical Sciences,
Babasaheb Bhimrao Ambedkar University (A Central University)
Vidhya Vihar, Raebareli Road
Lucknow-226 025 (UP), India

2. Department of Pharmacology,
College of Pharmacy
Prince Sattam Bin Abdulaziz University
Al-Kharj, KSA

3. Department of Pharmaceutical Sciences,
King Faisal University
AL-Ahsa, KAS

***Corresponding author**
Department of Pharmaceutical Sciences,
Babasaheb Bhimrao Ambedkar University (A Central University)
Vidhya Vihar, Raebareli Road
Lucknow-226 025 (UP), India
Email: gauravk@bbau.ac.in
+91 9670204349

Supplementary table 1: Treatment schedule

S. No.	Group	Treatment			
1.	Control	1 mM EDTA- saline, 2 ml/kg, s.c.			
2.	taVNS control	Pulse width -1.0 ms , Frequency-6.0 Hz, Voltage – 6v, Duration – 240 min.			
3.	DMH control	DMH (30 mg/kg/week, s.c.)			
**Transcutaneous Auricular Vagus Nerve Stimulation (taVNS)**		**Pulse width (ms)**	**Frequency (Hz)**	**Voltage (V)**	**Duration (Min.)**
4.	DMH + taVNS 1	1.2	4.0	6.0	33.33 min/week (200 min. for 6 weeks)
5.	DMH + taVNS 2	1.0	2.0	3.0	40.00 min/week (240 min. for 6 weeks)
6.	DMH + taVNS 3	1.0	6.0	6.0	40.00 min/week (240 min. for 6 weeks)
7.	DMH + taVNS 4	1.4	6.0	2.0	26.66 min/week (160 min. for 6 weeks)
8.	DMH + taVNS 5	1.6	1.0	5.0	20.00min/week (120 min. for 6 weeks)
9.	Standard chemotherapy	Leucovorin followed by 5-Flourouracil (25 mg/kg, i.p.) + DMH (30 mg/kg, s.c.)			
10.	Dummy control	Only stimulator without any current			

Supplementary table 2: Sequence of forward and reverse primers used for quantitative RT-PCR

S.No.	Primer	Sequence
**1.**	Bcl-2 F	GTGGATGACTGAGTACCTGAAC
**2.**	Bcl-2 R	GAGACAGCCAGGAGAAATCAA
**3.**	Bcl-xl F	CCCTCGTATCTGGAAGCCAC
**4.**	Bcl-xl R	CAGCGGAGACCTCGTTTTCT
**5.**	BAX F	TGCTACAGGGTTTCATCCAG
**6.**	BAX R	RGACACTCGCTCAGCTTCTT
**7.**	BAD F	CTCCGAAGAATGAGCGATGAA
**8.**	BAD R	ATCCCACCAGGACTGGATAA
**9.**	VDAC F	GGAGTTTGGTGGCTCCATTTA
**10.**	VDAC R	GACCTGATACTTGGCTGCTATTC
**11.**	Cytochrome - C F	TCCATTTCCCTTCCTTGGGC
**12.**	Cytochrome -C R	ATCGGGGCTGTCCAACAAAA
**13.**	Apaf-1 F	GAACATAGACTCCCGGGTAAAG
**14**	Apaf-1 R	CTTGTCTCCCAGACCCTTATTG
**15.**	Procaspase-9 F	GGCTCTCTGGCTTCATTCTT
**16.**	Procaspase-9 R	GGGTCCAGCTTCACTACTTTC
**17.**	α7nAchR F	AACTGGTGTGCATGGTTTCTGCGC
**18.**	α7nAchR R	AGATCTTGGCCAGGTCGGGGTCCC
**19.**	NFκB F	GGGCTACGAAGTCAAACCCA
**20.**	NFκB R	RTTCTCCTCAATCCGGTGACG
**21.**	TNF-α F	GCAGGTCTACTTTGGAGTCATT
**22.**	TNF-α R	GGCTCTGAGGAGTAGACGATAA
**23.**	HMGB-1 F	AAGACGACGAGGAGGATGAA
**24.**	HMGB-1 R	ACTGCGCTAGAACCAACTTATT
**25.**	β-actin F	TGCAGGATCGTGAGGAACAC
**26.**	β-actin F	AGCGTGATTGTAACGCCTGA

Supplementary table 3: Effect of taVNS on ECG against DMH induced colon carcinogenesis.

ECG Parameters	Control	taVNS control	DMH control	taVNS 1	taVNS 2	taVNS 3	taVNS 4	taVNS 5	Standard chemotherapy	Dummy control
RR Interval (s)	0.18±0.01[c]	0.17±0.01[c]	0.16±0.01	0.15±0.01[c]	0.17±0.01[c]	0.20 ±0.02[c]	0.17 ±0.01[c]	0.16±0.01[c]	0.16±0.01[c]	0.15±0.01[c]
Heart Rate (BPM)	330.84±24.81[a]	355.11±9.69	380.1±29.96	386.42±23.73	349.28±23	299.04±25.43[c]	357.16±28.5	374.01±29.92	373.3±17.13	411.46±25.66
PR Interval (s)	0.04±0.002	0.04±0.003	0.04±0.003	0.03±0.002[c]	0.04±0.001	0.04±0.002	0.04±0.003	0.04±0.003	0.04±0.001	0.03±0.002[c]
P Duration (s)	0.01±0.001	0.01±0.001	0.01±0.008	0.01±0.0001	0.01±0.001	0.01±0.001	0.01±0.001	0.01±0.001	0.01±0.006	0.01±0.007
QRS Interval(s)	0.01±0.008[c]	0.01±0.001[c]	0.02±0.001	0.02±0.001	0.02±0.001	0.02±0.001[c]	0.01±0.006	0.02±0.003	0.01±0.007[c]	0.01±0.0
QT Interval(s)	0.09±0.01[c]	0.05±0.003[c]	0.07±0.007	0.06±0.004	0.14±0.01[c]	0.16±0.01[c]	0.06±0.004	0.05±0.002[c]	0.05±0.003[c]	0.05±0.002[c]
J T Interval (s)	0.07±0.005[c]	0.03±0.002[c]	0.05±0.003	0.04±0.002[c]	0.03±0.002[c]	0.03±0.001[c]	0.04±0.002[c]	0.04±0.002[c]	0.03±0.002[c]	0.03±0.002[c]
Tpeak T end Interval (s)	0.04±0.003[c]	0.02±0.001[c]	0.03±0.002	0.03±0.001	0.02±0.001	0.03±0.002[c]	0.03±0.002[c]	0.02±0.001[c]	0.01±0.001[c]	0.02±0.001[c]
QTc (s)	0.19±0.01[c]	0.12±0.01[c]	0.15±0.01	0.16±0.01	0.14±0.01	0.16±0.01	0.16±0.01	0.12±0.01[c]	0.13±0.01	0.14±0.01
P Amplitude (mV)	0.1±0.01[c]	0.06±0.004[c]	0.04±0.003	0.08±0.006[c]	0.05±0.003[c]	0.09±0.01[a]	0.04±0.003	0.06±0.004[c]	0.04±0.003	0.05±0.003[a]
Q Ampli (mV)	0.04±0.002[c]	0.02±0.001[c]	0.20±0.01	0.03±0.002[c]	0.07±0.005[c]	0.02±0.001[c]	0.06±0.004[c]	0.03±0.001[c]	0.01±0.001[c]	0.07±0.005[c]
R Amplitude (mV)	1.80±0.15	1.17±0.09	1.35±0.12	1.17±0.09	0.24±0.02[c]	1.77±0.15	0.97±0.07	1.17±0.09	0.76±0.05	0.94±0.07
S Amplitude (mV)	-0.14±0.01[a]	-0.07±0.005[c]	-0.16±0.01	-0.05±0.003[c]	0.009±0.001[c]	-0.14±0.01[a]	-0.17±0.01	-0.07±0.001[c]	-0.13±0.01[c]	-0.12±0.01[c]
T Amplitude (mV)	0.40±0.02[c]	0.16±0.01[a]	0.12±0.01	0.30±0.02[c]	0.24±0.02[c]	0.38±0.02[c]	0.29±0.02[c]	0.16±0.01[a]	0.03±0.0001[c]	0.02±0.001[c]
ST Height (mV)	0.10±0.07[c]	0.01±0.001[c]	0.03±0.002	0.05±0.001[c]	0.09±0.001[c]	0.09±0.001[c]	0.04±0.003[c]	0.01±0.001[c]	0.05±0.003[c]	0.10±0.01[c]

(Values are presented as Mean ±SD), each group contains 8 animals. Comparisons were made on the basis of the one-way ANOVA followed by Bonferroni multiple test. All groups were compared to the toxic control group ([a]p < 0.05, [b]p < 0.01, [c]p < 0.001).

Supplementary table 4: Effect of taVNS on HRV against DMH induced colon carcinogenesis.

HRV	Control	taVNS control	DMH control	taVNS 1	taVNS 2	taVNS 3	taVNS 4	taVNS 5	Standard chemotherapy	Dummy control
**Time domain**										
**Average RR (ms)**	181.3[a]±14.78	168.90±12.16	157.80±9.15	155.27±9.64	171.8±4.40	200.64[c]±13.15	167.99±12.97	160.42±12.63	160.72±11.72	145.82±9.21
**Median RR (ms)**	182[a]±13.23	170.6±13.27	158.8±10.26	156.5±11.63	172±15.29	202.4[c]±12.37	169.16±12.40	161.00±14.28	162.8±13.11	147.2±12.20
**SD RR (ms)**	6.22±0.50[c]	9.18±0.85[c]	3.97±0.34	2.31±0.19[c]	8.60±0.75[c]	6.57± 0.45[c]	8.58±0.96[c]	7.20±0.62[c]	7.20±0.65[c]	3.36±0.27
**SDARR**	3.40±0.29[c]	9.44±0.83[c]	8.50±0.95	6.07±0.17[c]	21.74±1.93[c]	8.16±0.73[c]	21.60±1.97[c]	21.604±1.82[c]	17.83±1.29[c]	1.93±0.16
**CV RR (ms)**	0.04±0.002[c]	0.05±0.003[c]	0.02±0.001	0.01±0.001	0.04±0.003[c]	0.03±0.002[c]	0.09±0.01[c]	0.03±0.001[c]	0.03±0.002[c]	0.02±0.001
**Frequency domain**										
**LF(μs²)**	15.38±1.33[c]	0.14±0.01	1.2±0.10	0.27±0.02[c]	0.38±0.02	1.33±0.11[a]	9.17±0.79[c]	1.45±0.13[b]	0.64±0.05	0.82±0.06
**HF(μs²)**	60.84±5.95[c]	0.84±0.07[c]	9.10±0.81	1.33±0.12[c]	1.71±0.11[c]	5.25±0.35	18.94±1.56[c]	4.97±0.37[a]	6.23±0.51	7.48±0.65
**LF/HF**	0.25±0.02[c]	0.16±0.01	0.13±0.01	0.36±0.03[c]	0.21±0.01[c]	0.25±0.01[c]	0.48±0.03[c]	0.29±0.02[c]	0.10±0.001	0.10±0.01
**VLF (μs²)**	1.69±0.13[c]	2.77±0.25[c]	1.36±0.03	6.66±0.48[c]	3.12±0.27[c]	1.36±0.02	5.53±0.49[c]	1.38± 0.12[c]	1.70±0.13[c]	2.04±0.17[c]

(Values are presented as Mean ± SD), each group contains 8 animals. Comparisons were made on the basis of the one-way ANOVA followed by Bonferroni multiple test. All groups were compared to the toxic control group ([a]p < 0.05, [b]p < 0.01, [c]p < 0.001).

Supplementary table: 5 Effect of taVNS on weight against DMH induced colon carcinogenesis.

		Average Weight (gm)				Weight variation
**S.No.**	**Group**	**0 week**	**2 week**	**4 week**	**6 week**	**(%)**
1.	Control	61.64	71.00	73.37	78.25	21.22
2.	taVNS control	92.15	101	113	118.75	22.40
3.	DMH control	104.64	105.5	91.5	91.66	-14.17
4.	DMH+taVNS 1	112.27	125.25	140.25	138	18.65
5.	DMH+taVNS 2	82.73	101.14	100.85	104.66	20.95
6.	DMH+taVNS 3	82.68	105.2	115.8	109.66	24.42
7.	DMH+taVNS 4	77.34	94.66	110.83	105.66	26.80
8.	DMH+taVNS 5	91.43	111.5	126.16	131.00	30.20
9.	Standard chemotherapy	103.07	102.33	124.00	117.66	12.4
10.	Dummy control	98.27	110.75	119.25	130.75	24.84

(Values are presented as Mean ± SD), each group contains 8 animals. Comparisons were made on the basis of the one-way ANOVA followed by Bonferroni multiple test. All groups were compared to the toxic control group ($^{a}p < 0.05$, $^{b}p < 0.01$, $^{c}p < 0.001$).

Supplementary table 6: Quantification of H & E staining and scanning electron microscopy against DMH induced colon carcinogenesis

Group	Intensity (%)	
	H & E staining	SEM (500 X)
Control	25.22±2.16	27.09±1.94
taVNS control	46.48±3.12[c]	30.41±2.19
DMH control	29.07±2.42	32.41±3.26
taVNS1	28.87±2.67	31.54±2.89
taVNS 2	24.49±2.20	31.04±3.67
taVNS 3	25.82±2.90	31.97±3.46
taVNS 4	23.34±2.10[a]	31.09±2.67
taVNS 5	34.56±2.76[a]	31.83±3.45
Standard chemotherapy	29.02±1.98	31.74±3.10
Dummy control	24.24±2.65	31.41±2.54

(Values are presented as Mean ± SD), each group contains 8 animals. Comparisons were made on the basis of the one-way ANOVA followed by Bonferroni multiple test. All groups were compared to the DMH control group ([a]$p < 0.05$, [b]$p < 0.01$, [c]$p < 0.001$).

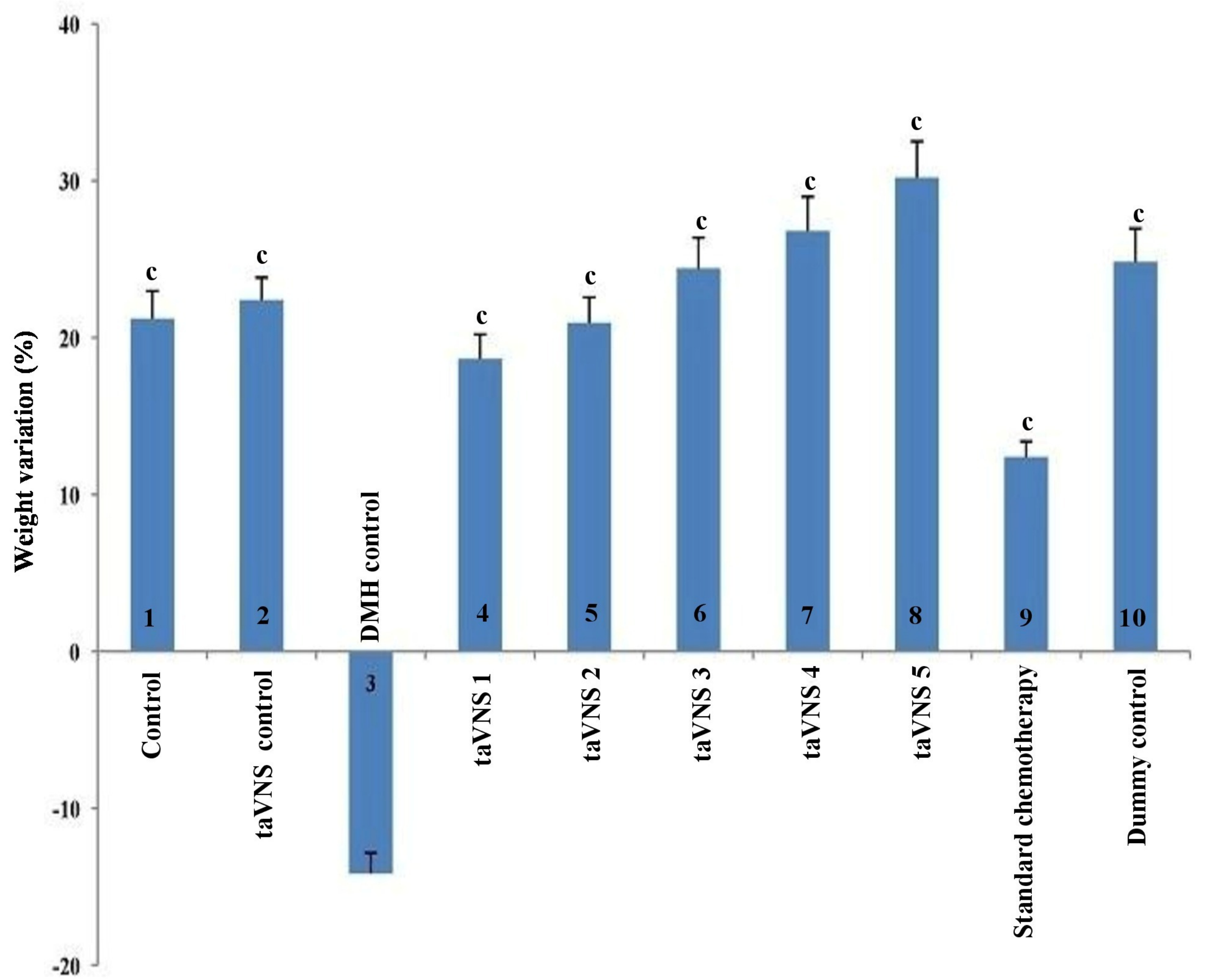

Weight variation (%)
40
30
20
10
0
-10
-20
c
1
2
3
4
5
6
7
8
9
10
Control
taVNS control
DMH control
taVNS 1
taVNS 2
taVNS 3
taVNS 4
taVNS 5
Standard chemotherapy
Dummy control

I

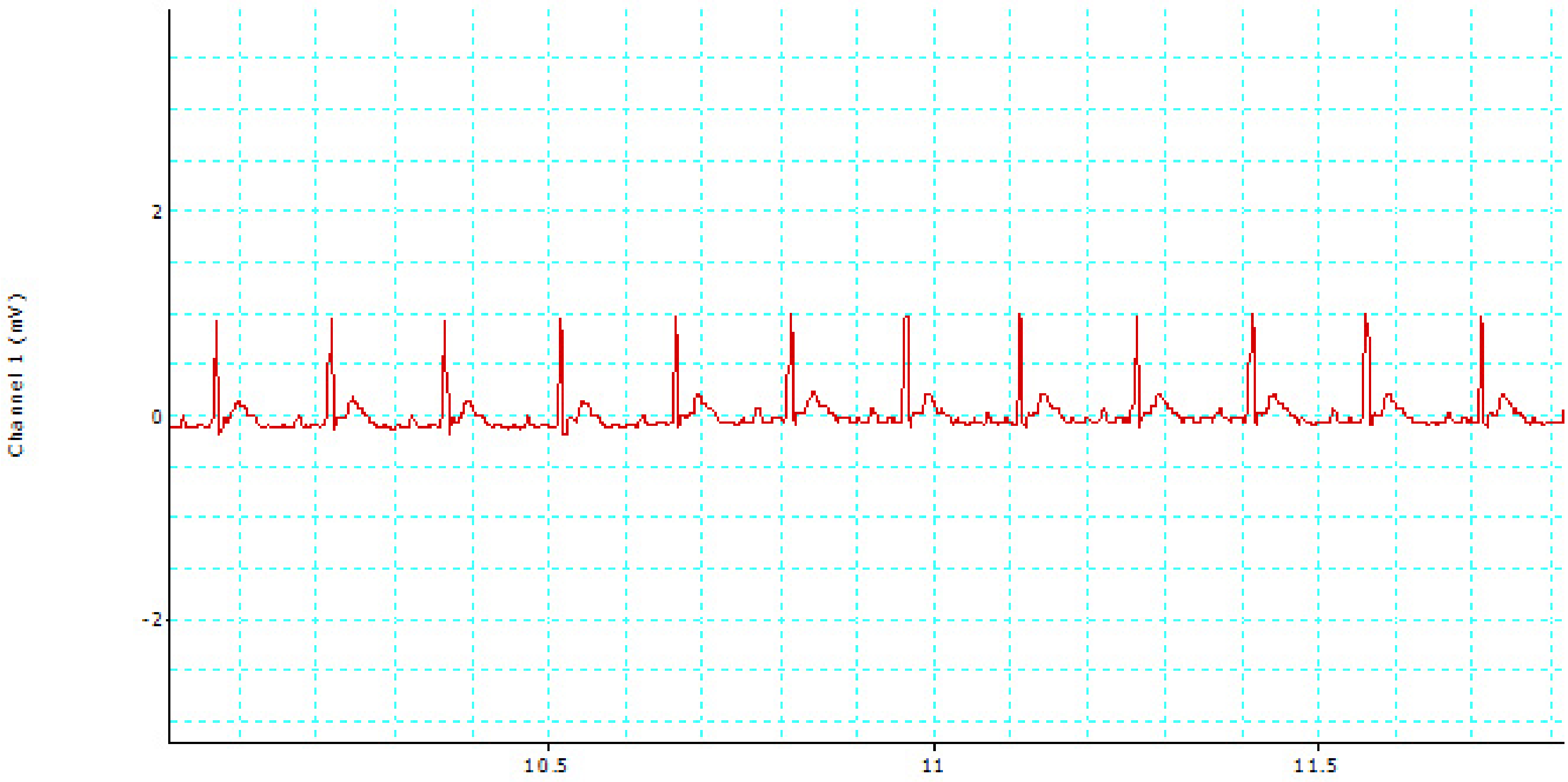

II

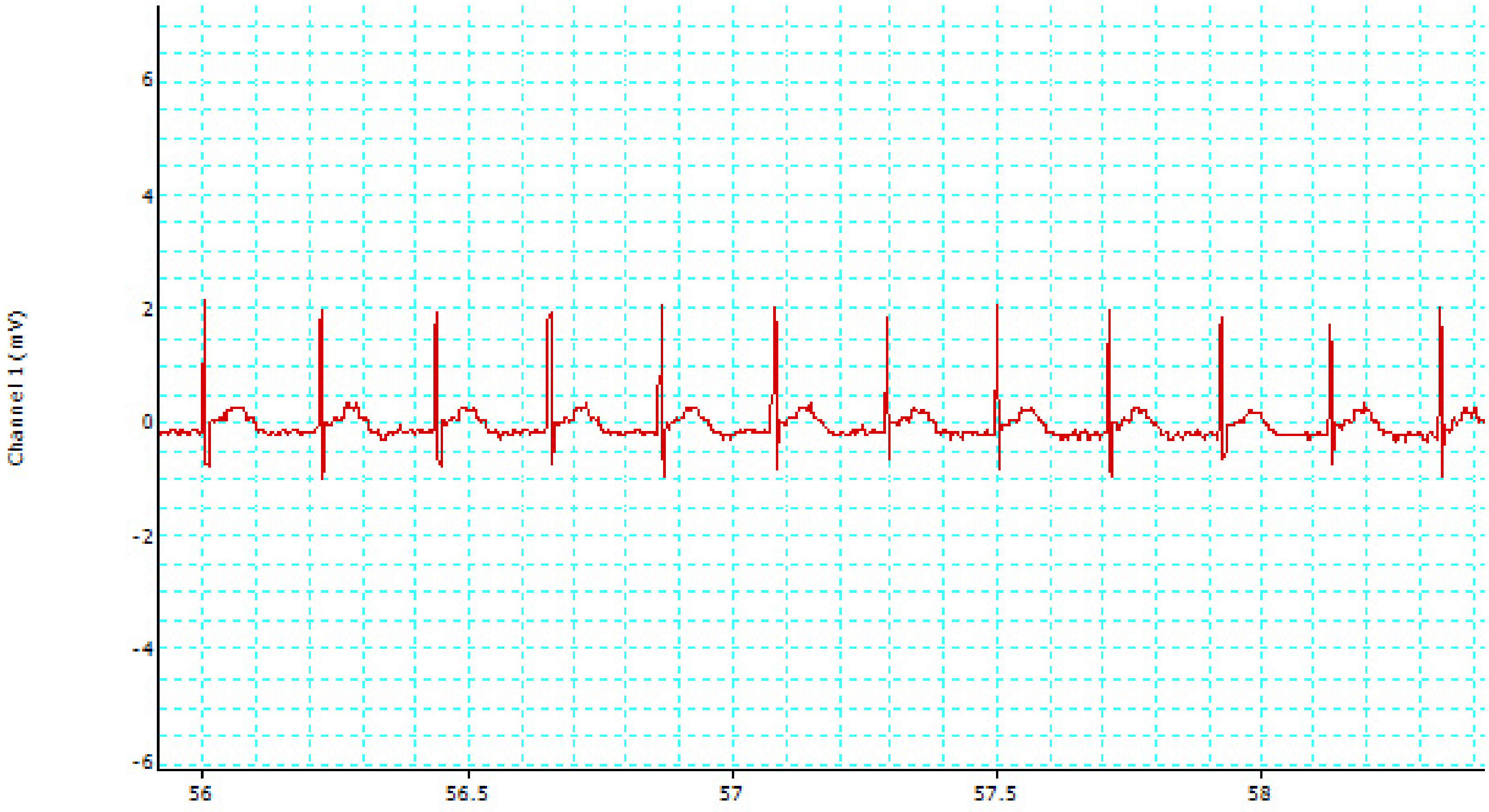

III

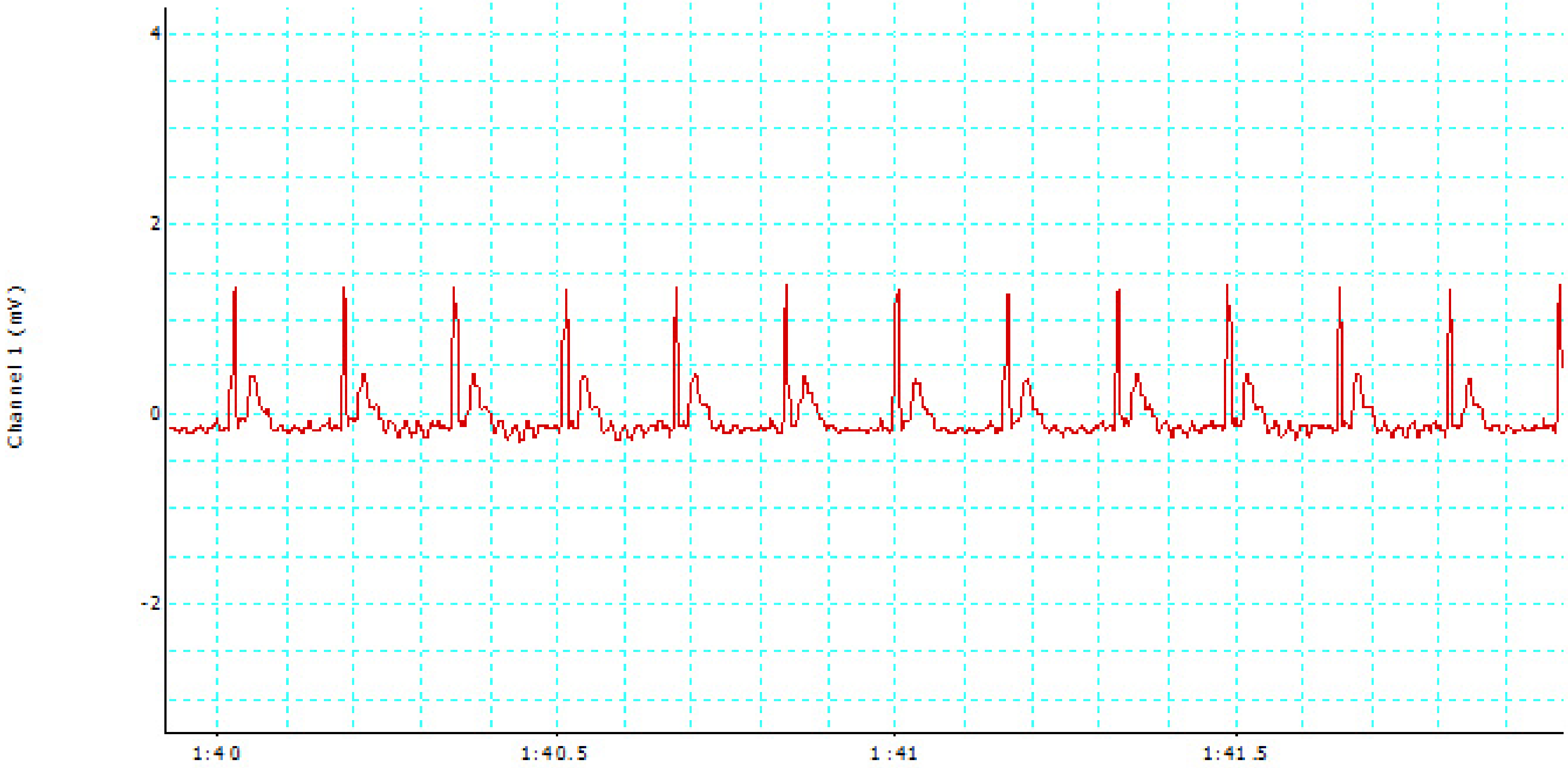

IV

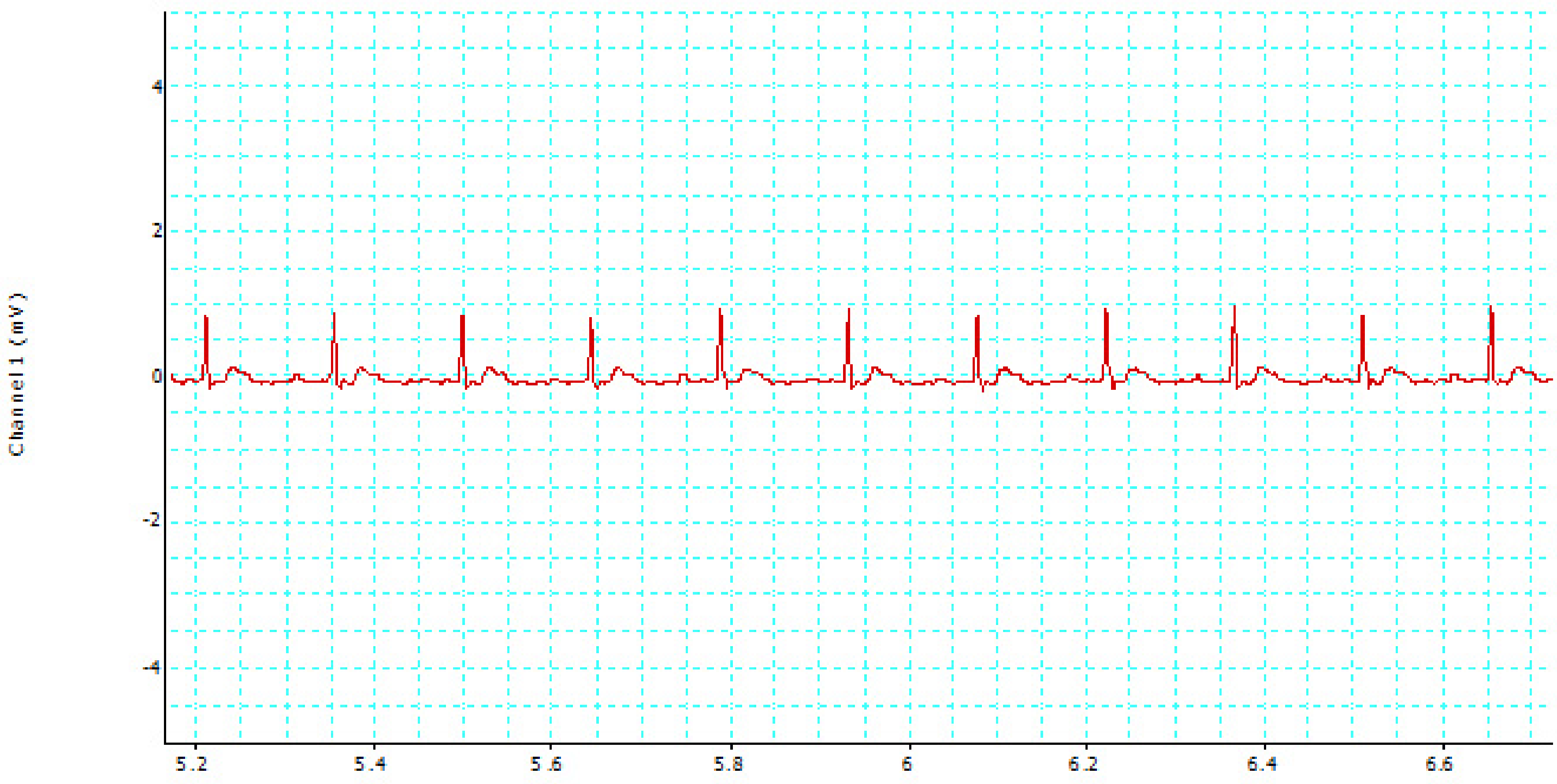

V

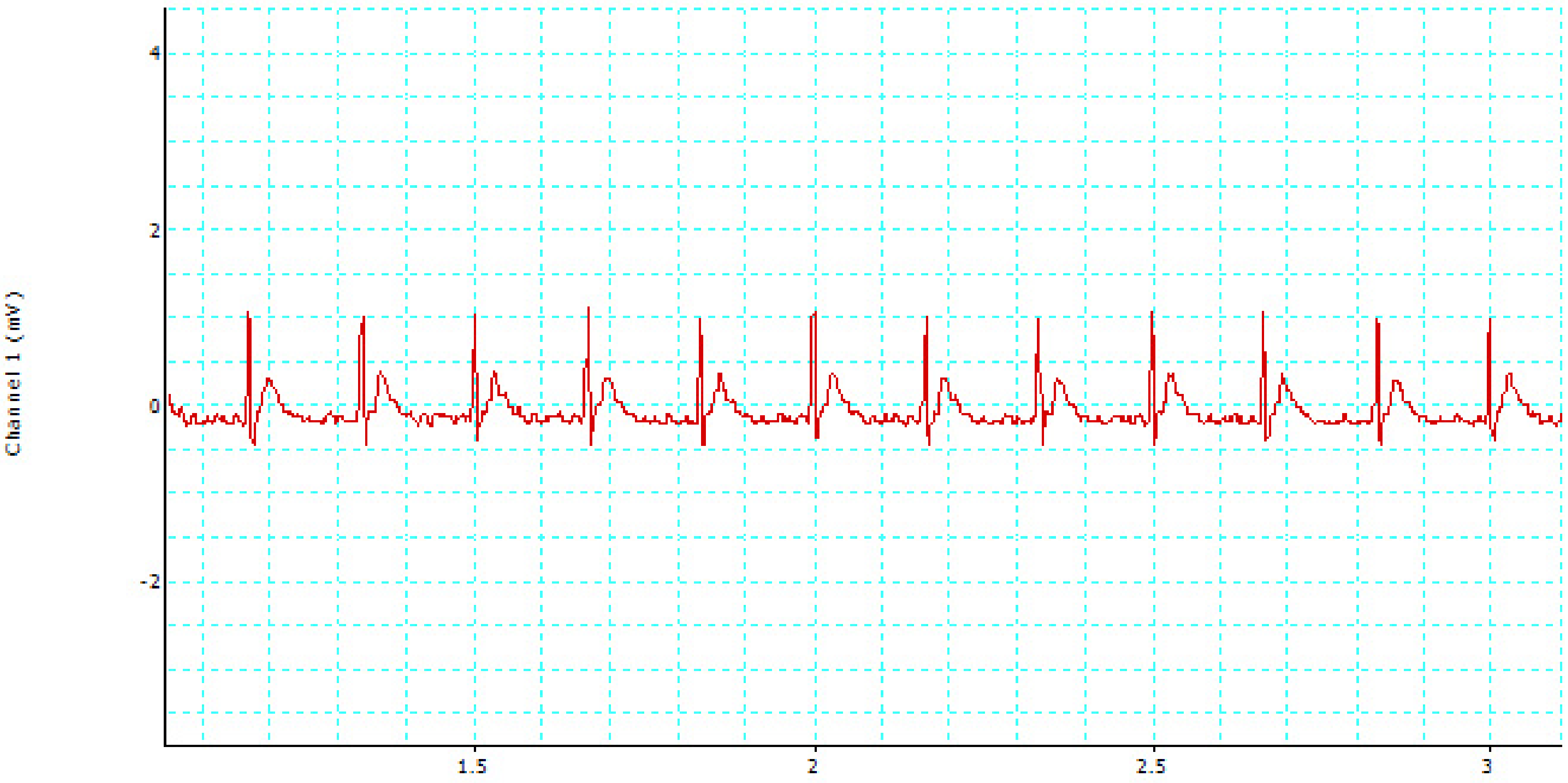

VI

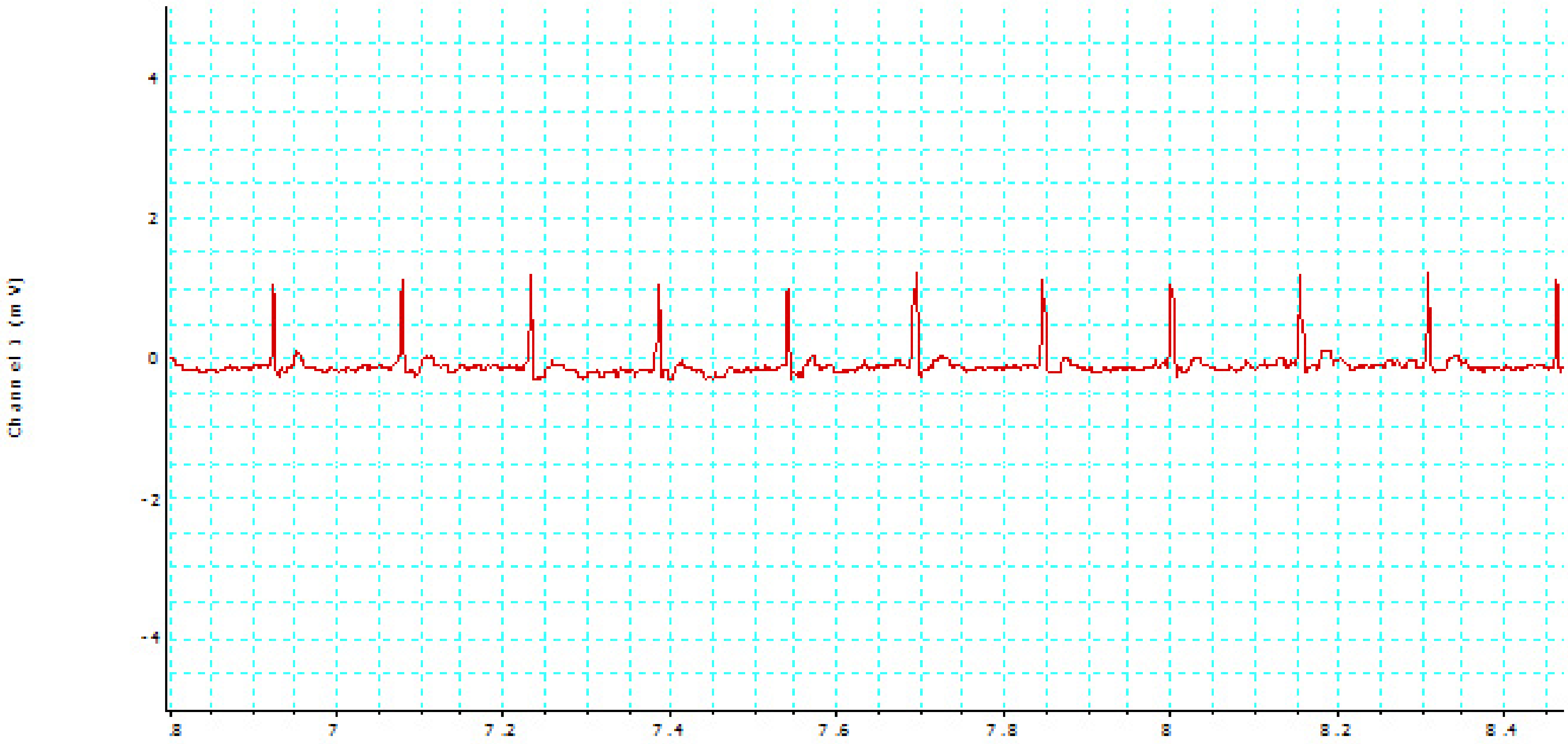

VII

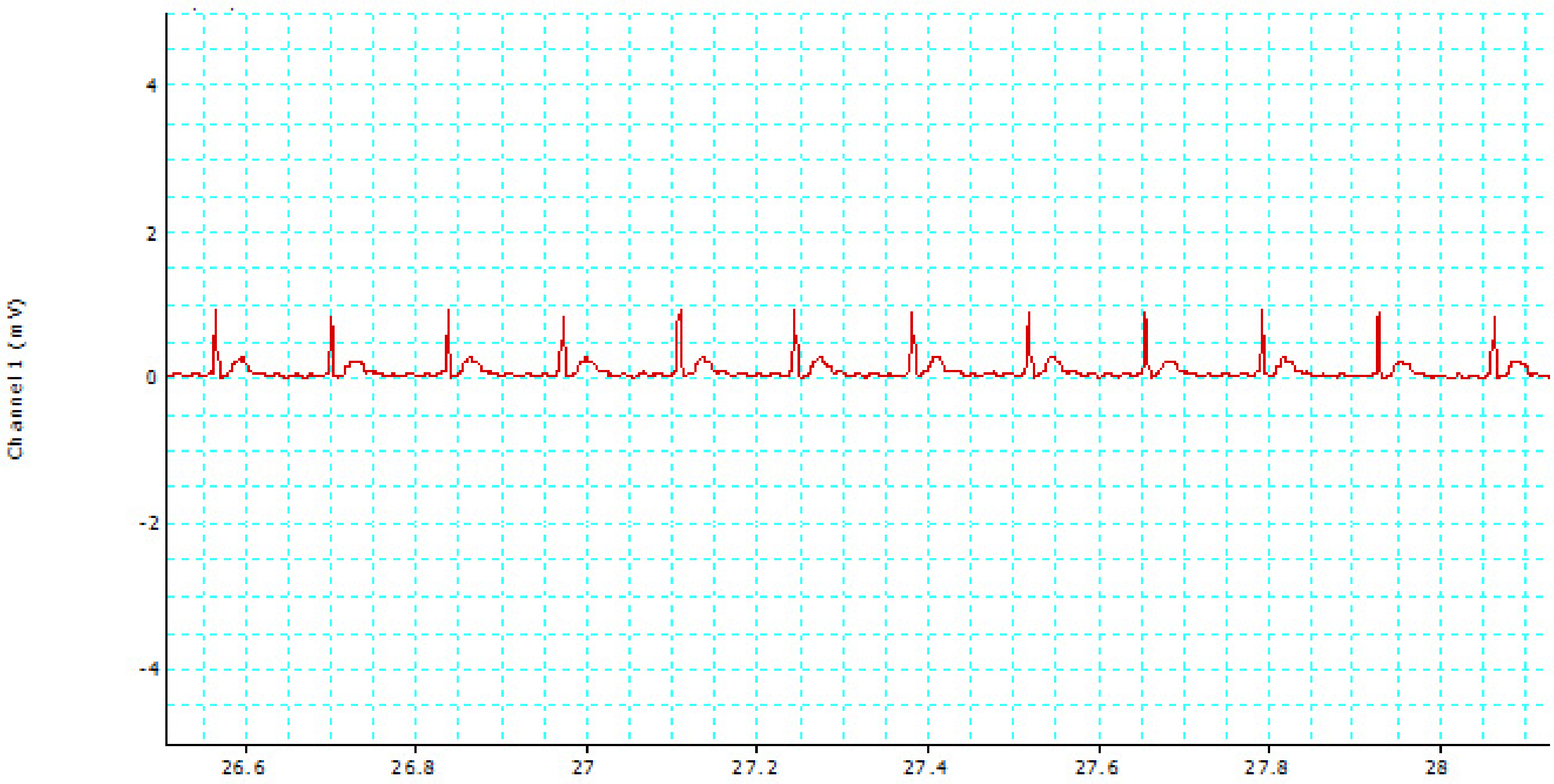

VIII

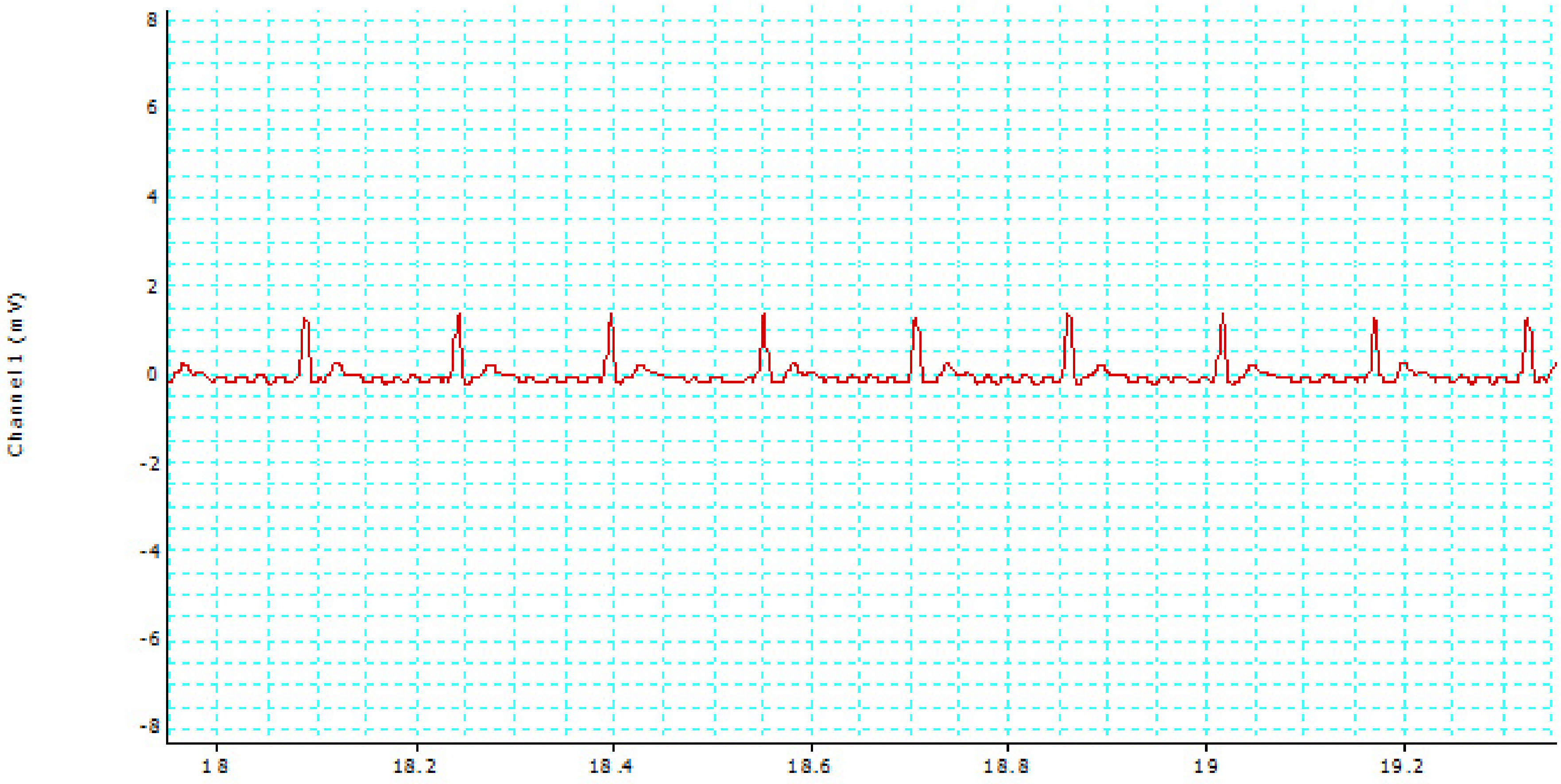

IX

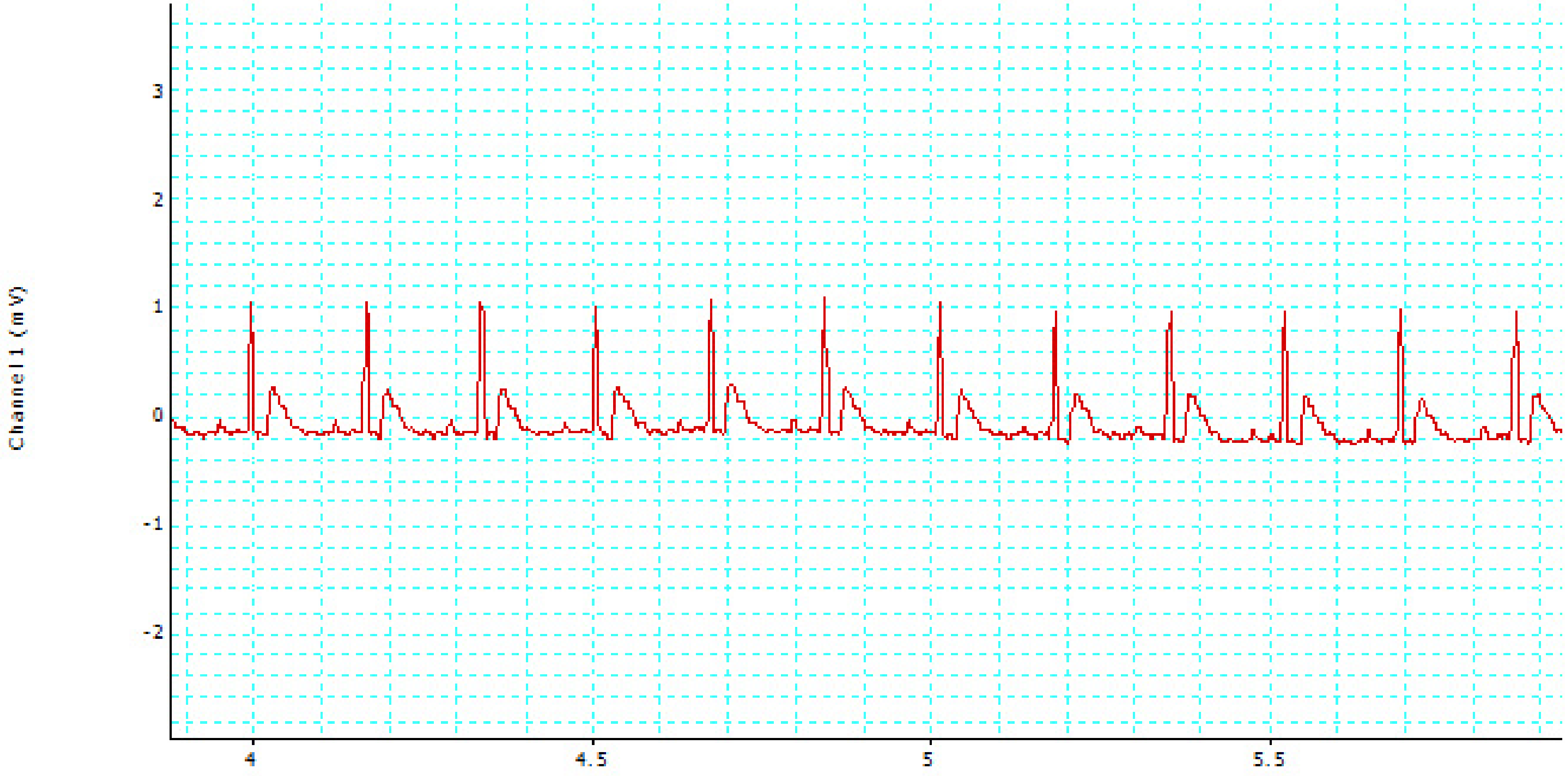

X

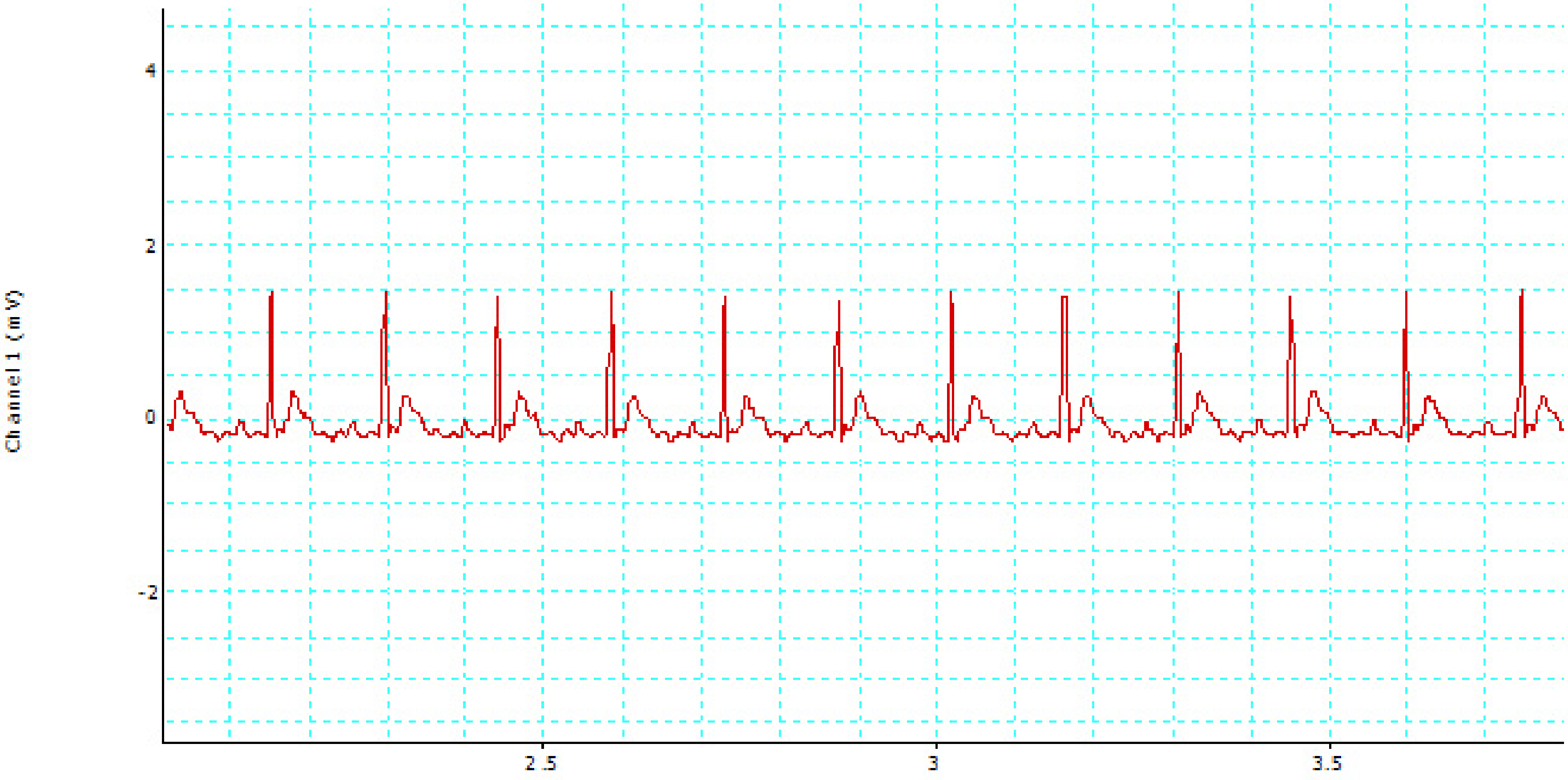

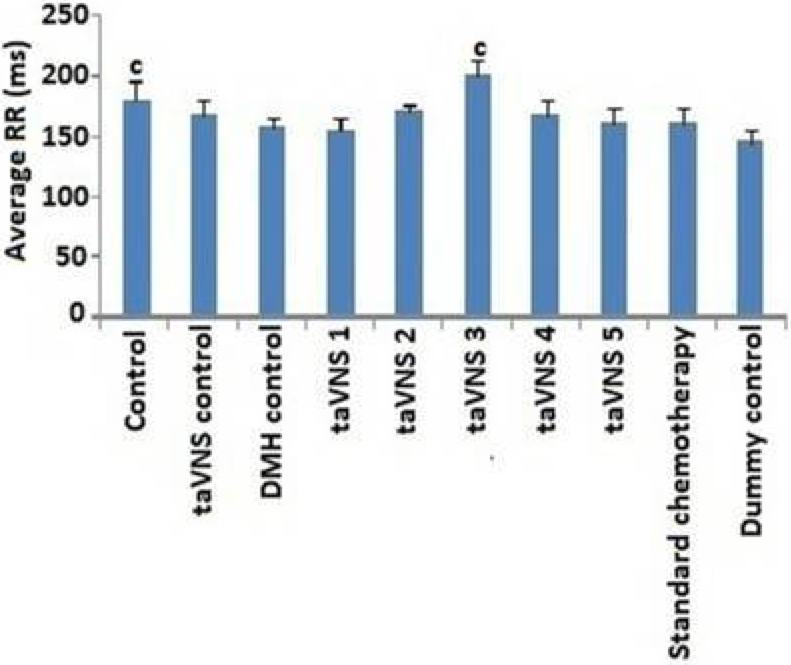

Average RR (ms)
250
200
150
100
50
0
c
c
Control
taVNS control
DMH control
taVNS 1
taVNS 2
taVNS 3
taVNS 4
taVNS 5
Standard chemotherapy
Dummy control

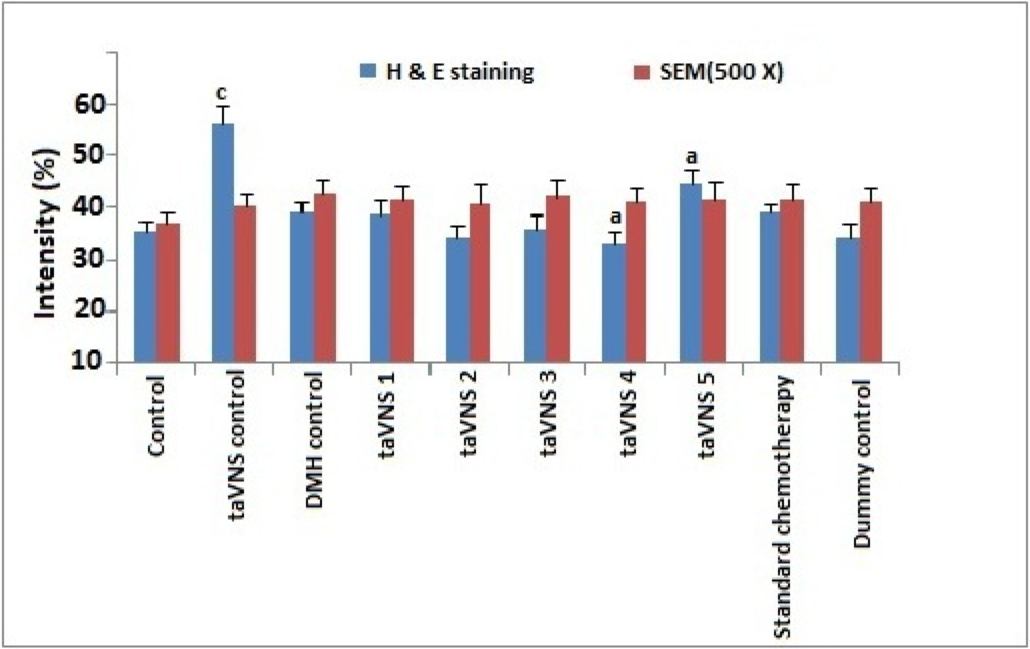

H & E staining
SEM(500 X)
Intensity (%)
60
50
40
30
20
10
c
a
a
Control
taVNS control
DMH control
taVNS 1
taVNS 2
taVNS 3
taVNS 4
taVNS 5
Standard chemotherapy
Dummy control

Figure Legends

Supplementary figure: 1 Effect of taVNS on weights against DMH induced colon carcinogenesis.

The details of groups as follows -
I-Control, II-taVNS control, III- DMH control, IV- taVNS1, V-taVNS 2, VI-taVNS 3, VII-taVNS 4, VIII-taVNS 5, IX- Standard chemotherapy, X- Dummy control.

Supplementary figure: 2 Representative recordings of ECG and HRV against DMH induced colon carcinogenesis.

The details of groups as follows -
I-Control, II-taVNS control, III- taVNS1, IV-taVNS 2, V-taVNS 3, VI- taVNS 4, VII-taVNS 5, VIII- Standard chemotherapy, IX- Dummy control, X- DMH control

Supplementary figure: 3 Effect of taVNS on RR interval against DMH induce colon carcinogenesis.

The details of groups as follows -
I-Control, II-taVNS control, III- DMH control, IV- taVNS1, V-taVNS 2, VI-taVNS 3, VII-taVNS 4, VIII-taVNS 5, IX- Standard chemotherapy, X- Dummy control.

Supplementary figure : 4 Quantification of H & E staining and scanning electron microscopy against DMH induced colon carcinogenesis

The details of groups as follows -
I-Control, II-taVNS control, III- DMH control, IV- taVNS1, V-taVNS 2, VI-taVNS 3, VII-taVNS 4, VIII-taVNS 5, IX- Standard chemotherapy, X- Dummy control.
